# pH-induced gene regulation of solvent production by *Clostridium acetobutylicum* in continuous culture: Parameter estimation and sporulation modelling

**DOI:** 10.1016/j.mbs.2012.11.004

**Published:** 2013-02

**Authors:** Graeme J. Thorn, John R. King, Sara Jabbari

**Affiliations:** a School of Mathematical Sciences, Mathematical Sciences Building, University of Nottingham, University Park, Nottingham NG7 2RD, UK; b Centre for Biomolecular Sciences, University of Nottingham, University Park, Nottingham NG7 2RD, UK

**Keywords:** AB fermentation, *Clostridium acetobutylicum*, Systems biology, ODE modelling

## Abstract

The acetone–butanol (AB) fermentation process in the anaerobic endospore-forming Gram-positive bacterium *Clostridium acetobutylicum* is useful as a producer of biofuels, particularly butanol. Recent work has concentrated on trying to improve the efficiency of the fermentation method, either through changes in the environmental conditions or by modifying the genome to selectively favour the production of one particular solvent over others. Fermentation of glucose by *C*. *acetobutylicum* occurs in two stages: initially the acids acetate and butyrate are produced and excreted and then, as the external pH falls, acetate and butyrate are ingested and further metabolised into the solvents acetone, butanol and ethanol. In order to optimise butanol production, it is important to understand how pH affects the enzyme-controlled reactions in the metabolism process. We adapt an ordinary differential equation model of the metabolic network with regulation at the genetic level for the required enzymes; parametrising the model using experimental data generated from continuous culture, we improve on previous point predictions (S. Haus, S. Jabbari, T. Millat, H. Janssen, R.-J. Fisher, H. Bahl, J. R. King, O. Wolkenhauer, A systems biology approach to investigate the effect of pH-induced gene regulation on solvent production by *Clostridium acetobutylicum* in continuous culture, BMC Systems Biology 5 (2011)) [1] both by using a different optimisation approach and by computing confidence intervals and correlation coefficients. We find in particular that the parameters are ill-determined from the data and that two separate clusters of parameters appear correlated, reflecting the importance of two metabolic intermediates. We extend the model further to include another aspect of the clostridial survival mechanism, sporulation, and by computation of the Akaike Information Criterion values find that the there is some evidence for the presence of sporulation during the shift.

## Highlights

► Metabolic ODE system as previously derived is underdetermined from the data. ► Inclusion of spore fraction post-pH shift in experiments improves the fit. ► Assuming approximately 75% of culture sporulates further improves the fit. ► Assays of acetyl-CoA, butyryl-CoA and spores will validate model.

## 1 Introduction

*Clostridium acetobutylicum* is an anaerobic endospore-forming Gram-positive bacterium that, under the correct conditions, can ferment hexose and other sugars into solvents such as acetone, butanol and ethanol (the acetone–butanol (AB) or acetone-butanol-ethanol (ABE) fermentation process) [2]. Recent work has concentrated on increasing the efficiency of fermentation by *C*. *acetobutylicum* in the production of butanol, in order to reduce the dependence on dwindling crude oil reserves and reduce biofuels’ impact on the environment by reducing crop biofuel production. Some other members of the *Clostridium* genus generate useful chemicals in much the same way as *C. acetobutylicum*, while some are highly pathogenic and cause devastating diseases (e.g. *Clostridium difficile* and *Clostridium botulinum*).

The metabolism of *C. acetobutylicum* has two distinct components—acidogenesis and solventogenesis. Initially, acids such as acetate and butyrate are formed from glucose and other hexose sugars, then, as the concentrations of acids increase, the metabolism shifts to produce solvents (acetone, butanol and ethanol—from which the process gets its name) from the acids, as well as producing spores. These latter processes are conjectured to be survival mechanisms in order for the organism to survive in more hostile conditions. The metabolic pathways leading to both acid and solvent production have been clearly defined and elucidated [2], with one of the primary drivers behind the shift from one to the other being the external pH [3].

**Fig. 1:**
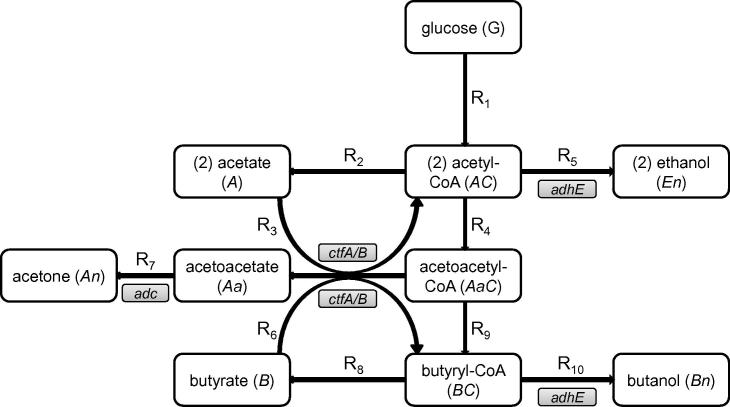
Diagram of the simplified *Clostridium acetobutylicum* metabolism network (after Haus et al. [1]), showing the ten reaction considered here, and the relevant genes encoding for the metabolic enzymes (G – glucose, AC – acetyl-CoA, A – acetate, AaC – acetoacetyl-CoA, BC – butyryl-CoA, B – butyrate, En – ethanol, Bn – butanol, Aa – acetoacetate, An – acetone, *ctfA*/*B* – CoA-transferase (with two subunits), *adc* – acetoacetate decarboxylase, *adhE* – aldehyde dehydrogenase). Note that the (2) denotes how many of the individual molecules are involved in each reaction, and that the gene marked *adhE* in this network is often called *adhE1*.

The reduced metabolism network introduced elsewhere [1] is shown in Fig. 1. Glucose is transported into the cell through a phosphoenolpyruvate-dependent phosphotransferase system, and is then glycolysed into pyruvate [2]. Acetyl-CoA (AC in our model) produced from this is a branch-point in the metabolic network, being the starting point of both the acidogenic (producing acetate (A) and butyrate (B)) and solventogenic (producing acetone (An), butanol (Bn) and ethanol (En)) pathways. In the acid producing branches, acetyl-CoA and butyryl-CoA (BC) are first converted into their respective phosphates, and then converted to acetate and butyrate [2]. These acids are then excreted from the cell.

Reinternalised butyrate and acetate can be converted to butyryl-CoA and acetyl-CoA respectively in Ping-Pong-Bi-Bi reactions, in the process converting acetoacetyl-CoA to acetoacetate. Both reactions are mediated by CoA-transferase, which has two subunits, CtfA/B [4]. The acetoacetate intermediate is then converted to acetone via the enzyme acetoacetate decarboxylase (Adc). Finally, acetyl-CoA and butyryl-CoA are converted to acetylaldehyde and butyraldehyde, respectively, as intermediates for ethanol and butanol. Two separate dehydrogenase activities mediate these reductions.

*C*. *acetobutylicum* can prioritise different pathways depending on external conditions, and can produce ethanol independently of acetone and butanol fermentation whilst in continuous culture—ethanol is produced at the same rate in both the acidogenesis and solventogenesis phases of the metabolism [1]. Experiments show how the external pH can trigger a shift in metabolism of *C*. *acetobutylicum*: cells primarily produce acids (acetate, butyrate) if the external pH is greater than 5 but produce primarily solvents (acetone, butanol) if the external pH is less than 5 [5–7,3].

Solventogenesis is postulated as part of a survival strategy [2], another part being the formation of endospores, the latter not being considered in the initial metabolism model adopted below. *C*. *acetobutylicum*, unlike some other bacteria, is unable to maintain its internal pH at a constant level, which makes it particularly susceptible to changes in the external pH [3]—instead it maintains a pH gradient across its membrane. Internal cellular pH is typically approximately one pH unit larger than the external pH, and this is maintained without significant delay under changes in the external conditions [3].

The study [1] from which our model is derived from (other models of clostridial metabolism for solvent production include that given in [8]) focussed on continuous culture experiments to measure changes in metabolism due to changes in pH, and fitted parameters to a combined metabolic, proteomic and genomic level model. There were three forward experiments (where the pH control was switched from high to low pH), and a single reverse experiment (where pH control was switched in the opposite direction). The parameter set was also used to study the effects of modifying gene regulation on the output production of the important chemical butanol. Our work builds on this, by using an improved optimisation method which has the flexibility of including all of the datasets generated, instead of a composite averaged dataset based upon all three forward experiments [1], and we perform post hoc analysis of confidence intervals and correlation coefficients to identify which parameters are well/badly determined from the data (Section 7), and suggest ways in which this determinability can be improved through further experimentation. We also consider the inclusion of another aspect of the survival strategy, sporulation (Sections 8–10), through the inclusion of simplified sporulation models and show this provides a better fit measured by residual sum of squares. As, in most cases, increasing the number of parameters in a model reduces the squared difference between the model and the experimental predictions [9], we compare the models using the Aikaike Information Criterion [9], and find that there is some evidence in the data to suggest that sporulation may be triggered in the continuous culture after the pH is shifted. Moreover, we derestrict the spore formation rate, which allows the spore fraction to reach up to 75% and show that this causes the *AIC* to fall below that of all models considered so far, but to a non-physical situation where less than 30% of the cells metabolise post-shift, which is not seen in the experiments. To improve the fit, reduce the parameter ranges found, and break some of the correlations, we recommend that for further continuous culture experiments, concentrations of the important intermediates acetyl-CoA and butyryl-CoA need to be measured, as well as a spore fraction assay.

## 2 Experimental method

We use experimental data generated for a previous study [1], where the organisms were grown in continuous phosphate-limited glucose-rich cultures with pH control. The details are provided elsewhere [1], so we run through the main points quickly here.

• *C*. *acetobutylicum* strain ATCC824 is grown under anaerobic conditions at 37 °C, and the precultures are prepared as previously described.
• The culture is kept phosphate-limted in a chemostat (BiostatB 1.5 l fermenter system from BBI, Melsungen, Germany), with 0.5 mM KH_2_PO_4_ and 4% (w/v) glucose in the medium and a dilution rate of $D=0.075\;{\text{h}}^{-1}$.
• The external pH is kept constant at pH 5.7 and 4.5 by automatic addition of 2 M KOH.
• Three forward shift experiments are performed by shifting the culture from pH 5.7 to 4.5, and one reverse shift experiment is performed by shifting the culture from pH 4.5 to 5.7:
  – ‘Forward 1’: acidogenesis was maintained for 137 h, then the pH control was stopped, allowing the metabolic shift to occur. 22 h after the removal of the pH control the shift had completed, and final measurement was taken at 215 h.
  – ‘Forward 2’: as in ‘Forward 1’—the pH control was switched off after 137.5 h, the metabolic shift occurred over 33.5 h and steady state was reached at approximately 236 h.
  – ‘Forward 3’: as in the previous two, but the pH control was switched off at 121 h, the metabolic shift lasted 29 h and the final measurement was taken at 215 h.
  – ‘Reverse’: the pH was controlled via the addition of KOH so that the change was in the opposite direction—the pH was initially kept low for 129 h, and then increased over 17 h to the high level.

The experimental data are given in the Supplementary material.

## 3 Metabolic model details

Following the previous work [1], the ten reactions in the metabolic model occur at the following rates $R_{1}\text{,}\ldots \text{,}R_{10}$ (the symbols represent the total intracellular and extracellular concentrations of the various chemicals given in Fig. 1, with the exception of the symbols Cf,Ad and *Ah*, which represent intracellular concentrations of the enzymes CtfA/B, Adc and AdhE, respectively):

(3.1) $$\begin{array}{cc} & R_{1}=\frac{2V_{1}\cdot G}{K_{1}+G}\text{,}\;R_{5}=\alpha_{5}\cdot \mathrm{AC}\cdot \mathrm{Ah}\text{,}\;R_{9}=\alpha_{9}\cdot \mathrm{BC}\cdot \mathrm{Ah}\text{,}\\ & R_{2}=\frac{V_{2}\cdot \mathrm{AC}}{K_{2}+\mathrm{AC}}\text{,}\;R_{6}=\alpha_{6}\cdot B\cdot \mathrm{AaC}\cdot \mathrm{Cf}\text{,}\;R_{10}=\frac{V_{10}\cdot \mathrm{AaC}}{K_{10}+\mathrm{AaC}}\text{.}\\ & R_{3}=\alpha_{3}\cdot A\cdot \mathrm{AaC}\cdot \mathrm{Cf}\text{,}\;R_{7}=\alpha_{7}\cdot \mathrm{Aa}\cdot \mathrm{Ad}\text{,}\\ & R_{4}=\frac{V_{4}\cdot \mathrm{AC}}{2(K_{4}+\mathrm{AC})}\text{,}\;R_{8}=\frac{V_{8}\cdot \mathrm{BC}}{K_{8}+\mathrm{BC}}\text{,} \end{array}$$

where Michaelis–Menten kinetics are assumed, except where enzyme concentrations are included directly. As per the previous work, all enzyme-mediated reactions ($R_{i}$) are assumed quasisteady: the $\alpha_{i}$ coefficients are defined for these reactions as $\alpha_{i}=k_{1i}k_{2i}/(k_{-1i}+k_{2i})$ where $k_{1i}$ is the rate of formation of the complex, $k_{-1i}$ is the degradation rate, and $k_{2i}$ is the reaction rate for production of the final products. In the above equations $V_{i}$ represents the maximum limiting rate of reaction *i*, and $K_{i}$ is the half-constant in the Michaelis–Menten expression. As in Haus et al. [1], we include stochiometric constants of 2 in $R_{1}$ and 0.5 in $R_{4}$ due to two molecules of acetyl-CoA being formed from one glucose molecule, and one acetoacetyl-CoA molecule formed from two acetyl-CoA. Note, however, that the stochiometry needs to be corrected from the previous work [1] where no account is taken of the number of molecules generated in reaction 4. This also affects the resulting ordinary differential equation metabolic model from that paper which is now as follows:

(3.2) $$\begin{array}{cc} & \frac{\text{d}\mathrm{AC}}{\text{d}t}=R_{1}-R_{2}+R_{3}-2R_{4}-R_{5}-D\cdot \mathrm{AC}\text{,}\;\frac{\text{d}\mathrm{BC}}{\text{d}t}=R_{10}-R_{8}+R_{6}-R_{9}-D\cdot \mathrm{BC}\text{,}\\ & \frac{\text{d}A}{\text{d}t}=R_{2}-R_{3}-D\cdot A\text{,}\;\frac{\text{d}B}{\text{d}t}=R_{8}-R_{6}-D\cdot B\text{,}\\ & \frac{\text{d}\mathrm{En}}{\text{d}t}=R_{5}-D\cdot \mathrm{En}\text{,}\;\frac{\text{d}\mathrm{An}}{\text{d}t}=R_{7}-D\cdot \mathrm{An}\text{,}\\ & \frac{\text{d}\mathrm{AaC}}{\text{d}t}=R_{4}-R_{3}-R_{6}-R_{10}-D\cdot \mathrm{AaC}\text{,}\;\frac{\text{d}\mathrm{Bn}}{\text{d}t}=R_{9}-D\cdot \mathrm{Bn}\text{.}\\ & \frac{\text{d}\mathrm{Aa}}{\text{d}t}=R_{3}+R_{6}-R_{7}-D\cdot \mathrm{Aa}\text{,} \end{array}$$

with the outflow terms representing dilution being the product of the dilution rate with the concentration of the metabolite. There are also three equations relating to the enzyme concentrations—these represent elevated enzyme production below some threshold pH, expressed via a function F(p):

(3.3) $$\begin{array}{cc} & \frac{\text{d}\mathrm{Ad}}{\text{d}t}=r_{\mathrm{Ad}}+r_{\mathrm{Ad}}^{+}\cdot F-D\cdot \mathrm{Ad}\text{,}\\ & \frac{\text{d}\mathrm{Cf}}{\text{d}t}=r_{\mathrm{Cf}}+r_{\mathrm{Cf}}^{+}\cdot F-D\cdot \mathrm{Cf}\text{,}\\ & \frac{\text{d}\mathrm{Ah}}{\text{d}t}=r_{\mathrm{Ah}}+r_{\mathrm{Ah}}^{+}\cdot F-D\cdot \mathrm{Ah}\text{.} \end{array}$$

The function F(p) is a smoothed step function ranging from 0 to 1 below a threshold pH level $p^{*}$ (again, a factor 1/2 was omitted from the previous work [1] so that their F(p) could range from 0 to 2; we correct this):

(3.4) $$F(p)=\frac{1}{2}\left(1-\tanh\left(n\left[p-p^{*}\right]\right)\right)\text{.}$$

(We note that the two corrections we have made to the model do not alter the qualitative conclusions of that work [1]: the first results in new parameters (given in the following table) while *F* in that paper never increased beyond one so the omission was of no consequence.) For each experiment, the function p(t) of the form

(3.5) $$p(t)=5.7-c_{1}\cdot \tanh(c_{2}\cdot c_{3})+c_{1}\cdot \tanh(-c_{3}[t-c_{2}])$$

is fitted to the pH data within each numerical simulation using the function nlinfit in Matlab. Eqs. (3.1)–(3.5) form the model used to fit the experimental data as described in the next section.

## 4 Choice of datasets for the parameter estimation

As there are four experimental datasets from which we could estimate the parameters, we have chosen three separate ways of combining the data for comparison of parameter estimates (note that this is distinct from the previous study [1], where the three forward experiments were used for fitting, and the reverse experiment was used for validation of the model).

• As in the previous work [1], we take an average data set based upon the three forward experiments. The data for experiments ‘forward 1’ and ‘forward 3’ are time-stretched so that the three phases (acidogenesis, the dynamic pH shift and solventogenesis) all occur over the same times as experiment ‘forward 2’, i.e. the start of solventogenesis is 33.5 h after the start of the dynamic shift phase for all time-stretched data sets. All three transformed datasets are then interpolated at identical time points using cubic splines, then averaged to provide a single high-time-density experiment’s worth of data.
• A dataset consisting of the three forward experiments, starting at the relevant initial conditions for each experiment and using all available data from these experiments.
• A dataset consisting of all available data from the four experiments (three forward, one reverse).

The first dataset generated from the experimental data was used in order that a direct comparison with previous work can be made. This will allow us to validate the averaged approach taken in the previous work [1], as the three forward experiments are not direct replications of each other. There is a possibility that interpolating the data and averaging introduces artefacts in the data, but if the reactions occur on time scales much shorter than the pH shift, then the pH can be considered to be almost constant as the reactions take place. Without a priori knowledge of the reaction rates, however, we are unable to compare the time scales directly, so we are comparing this method against the other two ways of treating the data.

Many optimisation methods extremise a given non-linear function, which for model-fitting is generally taken to be minimising the sum of the squared differences between the data and the numerical results, and so can easily take account of the different experiments (with their differing initial conditions) used in the datasets.

## 5 Optimisation details

In this section we will sketch out the method used for parameter fitting. There are 23 parameters over which we wish to optimise (for the original network: there are more parameters when we consider the sporulation-linked model), and as each one is restricted to being positive, we optimise using the logarithms (to base 10) of the parameters. This has the side-effect that the logarithms of the previously estimated parameters (Table 1 and [1]) are all of the same order of magnitude. We scale the logarithms further by restricting our search space so that they lie between an upper and a lower bound (set to −6 and +6 respectively for all parameters $V_{k}\text{,}\;K_{k}\text{,}\;\alpha_{k}\text{,}r_{x}$ and $r_{x}^{+}$ corresponding to an interval of $\left[10^{-6}\text{,}10^{6}\right]$; the bounds for *n* and $p^{*}$ were set at [10, 1000] and [4.5, 5.7]) and set the scaled bounds to [0,1]. The different limits for *n* and $p^{*}$ are to restrict the parameters to sensible values (particularly for the value at which the pH shift occurs—it is restricted to lying within the pH range of the experiments). It is known that the simplex algorithm (which we will employ) works best when the parameters over which to optimise have the same scale [10], and this choice of scaling means we optimise over the unit 23-(or more)-dimensional cube for the scaled transformed parameters.

The main optimisation method is a modified simplex method [11] which has all the features of simulated annealing at the start of the optimisation, and has the downhill local-search of the Nelder–Mead simplex method at the end. Once this algorithm terminates, a further round of Nelder–Mead simplex searching (using the minimum as a starting point) is used to tidy up the minimum found. The details of the modified simplex method can be found in the previous reference [11], but we provide our implementation of the method in the Supplementary material for completeness. As the method is a stochastic optimisation method, we repeat this starting from different uniformly generated starting points to generate fifty different sets of optimising parameters, and the lowest scoring sets are used to give the parameter estimates.

## 6 Calculation of confidence intervals and correlation coefficients

Once a parameter set $x^{*}$ is found, we can calculate post hoc confidence intervals and estimate the correlation coefficients between parameters. The method for this post hoc analysis follows that in [12] (and is summarised in the Supplementary material): under the assumptions that all errors between experimental data and the numerical prediction are independent and caused primarily by measurement error, minimising the weighted least squares difference (where the weights are $1/\sigma_{y}$, the inverse of the standard deviation of the error at each point) gives the maximum likelihood estimator (MLE) of the parameters. If the errors are all considered equal, then minimising the ordinary least squares (OLS) distance gives the MLE. Of course, if there are features not included in the model, then at least some of the errors are systematic and model-dependent, but we cannot account for these directly; we can, however, consider improvements to the model which would be reflected in smaller OLS values, such as including sporulation (see Section 8) or more steps in the metabolism.

We can use the OLS score function to compute likelihood ellipsoids, from which we calculate the dependent and independent confidence intervals, and estimated correlation coefficients. The dependent confidence interval assumes that the other parameters are exactly determined, and the independent confidence interval assumes that all parameters are equally uncertain. Geometrically, the dependent confidence interval is the intersection of the ellipsoid with the line given by the other parameters being fixed at their MLEs, and the independent confidence interval is the projection of the ellipsoid onto the coordinate axis.

## 7 Results

### 7.1 Parameter estimates

**Fig. 2:**
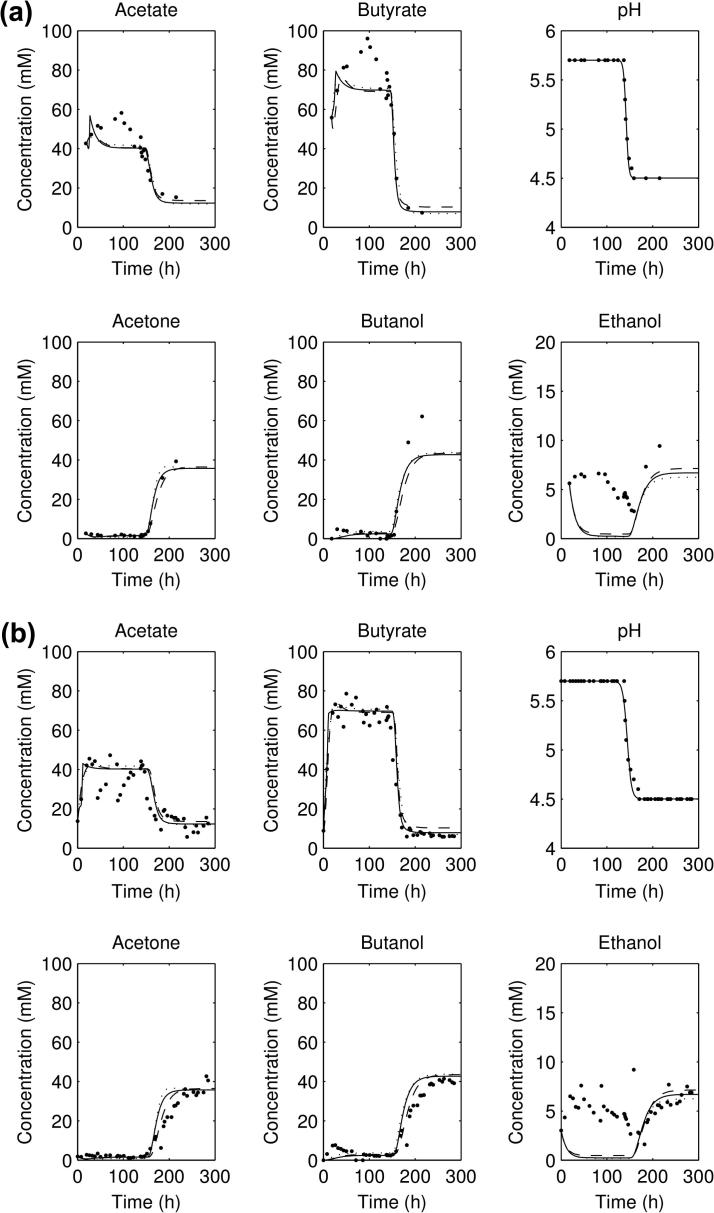
Plots of the acid and solvent concentrations from experimental data and simulated, using the (solid line) parameters from fitting to the three forward experiments; (dotted) parameters from fitting to the averaged data and (dashed) parameters from fitting to all four experiments: (a) first forward experiment and (b) second forward experiment.

**Fig. 3:**
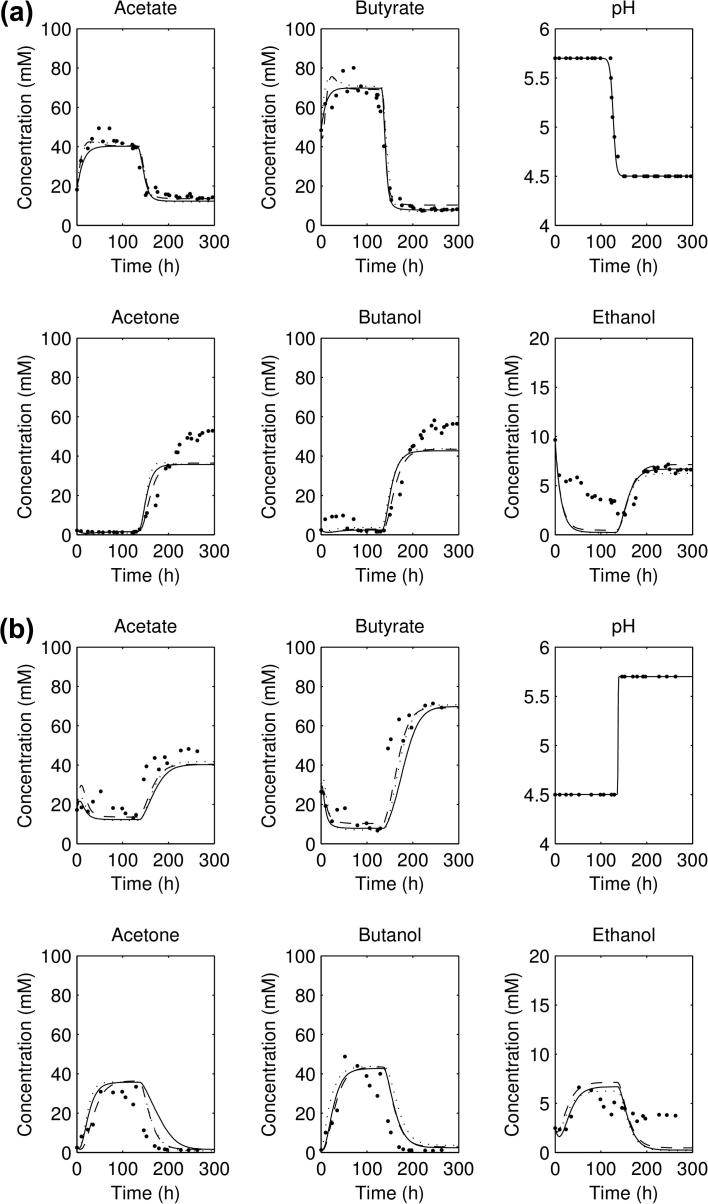
Plots of the acid and solvent concentrations from experimental data and simulated, using the (solid line) parameters from fitting to the three forward experiments; (dotted) parameters from fitting to the averaged data and (dashed) parameters from fitting to all four experiments: (a) third forward experiment and (b) reverse experiment.

The best fits for the three datasets are compared against each other in Figs. 2 and 3a. For the three forward experiments (Figs. 2 and 3a), the parameter sets all show an initial acidogenesis phase, followed by a switch to solventogenesis and acid consumption as the pH falls; for the reverse experiment (Fig. 3b) there is an initial solventogenesis phase, followed by a switch to acidogenesis and a fall in solvent concentrations as solvents are washed out. All simulations of the parameter sets plotted reach a steady state before the shift, and a steady state post-shift, but do not capture the exact shape of the ethanol concentration curve. It can be noticed that the exact concentrations of acetate and butyrate in the three forward experiments (pre-shift) are not captured accurately nor are the solvent concentrations post-shift.

The predictions from the lowest scorers of the three different selections of experimental data in some way validates the approach used in the previous work [1] of taking the averaged and interpolated dataset, as if the three experiments were exact repetitions of each other. (Note that we take the corrected parameter values provided by the authors through personal communication for the corrected model in our comparisons—see Table 1.) This would not hold if the experiments chosen to average were drastically different, but in this case the time taken for the shift in the pH of the three forward experiments is approximately the same (between 22 and 34 h), so the procedure turns out to be valid. The results from the new parameter fits still have the same issues as the original parameter set—there is still no fit at early times for the acid concentrations and there is still no ethanol production during the acidogenesis phase in the forward experiments (Figs. 2 and 3a). In fact, the concentrations of acetate and butyrate simulated are below those measured experimentally. This could indicate that there are some significant features which are missing from the model: for instance, metabolising the glucose into acetyl-CoA takes five steps rather than the one considered in the model [2], and involves at least three more enzymes. However, as the concentrations of these intermediates are not measured, introducing extra steps into the model may lead to overfitting (and more opportunities for correlated reaction rates) with the extra parameters not being determinable. Secondly, ethanol underproduction during acidogenesis from this model has been suggested [1] to be due to not considering the *adhE2* gene which is antagonistically regulated with *adhE1* (with only the latter being included in the model). *adhE1* is expressed during solventogenesis and so *adhE2* is downregulated here, but *adhE2* is upregulated during acidogenesis and could be responsible for ethanol production at the higher pH values.

**Fig. 4:**
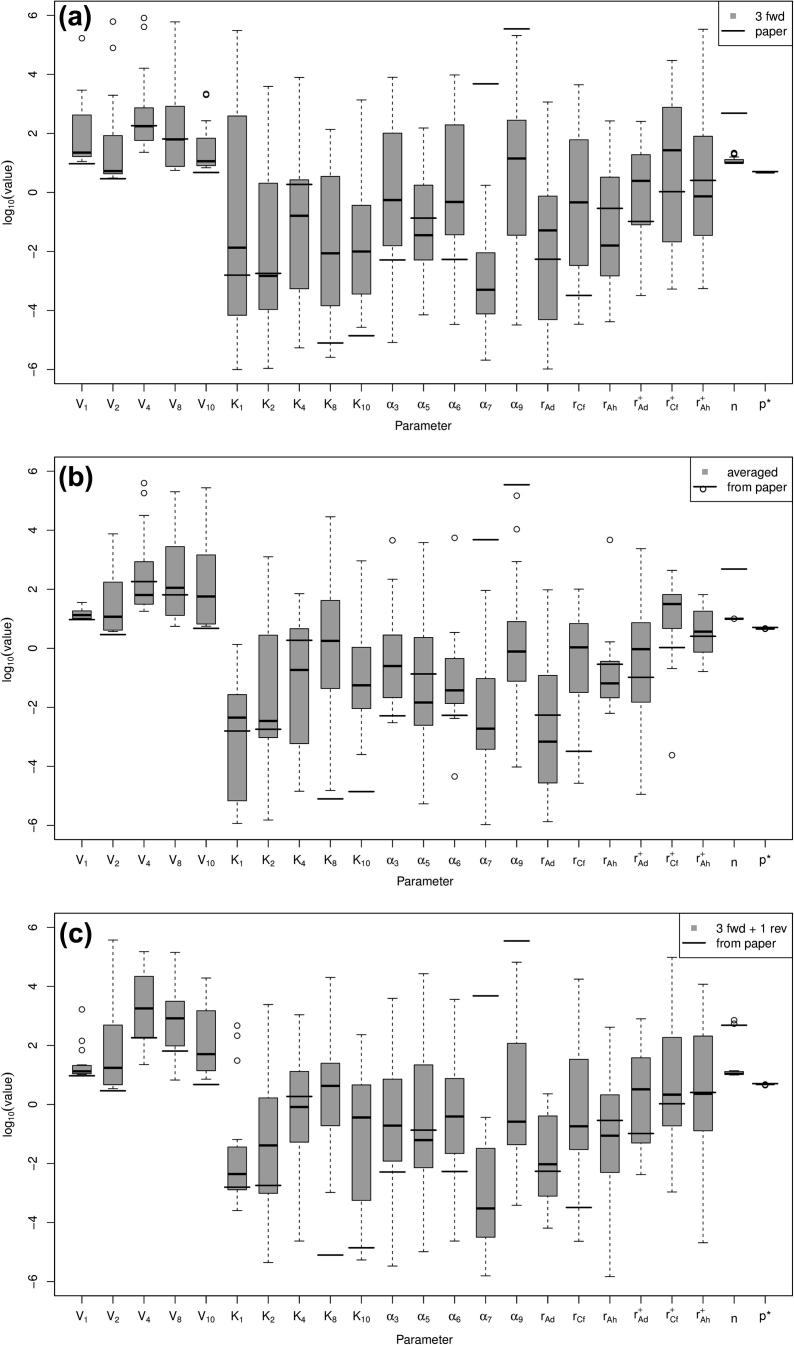
Boxplot of $\log_{10}(\text{parameters})$ for fitted parameter for the three ways of organising data: (a) all three forward experiments; (b) averaged data set and (c) all four experiments. Parameters fitted elsewhere [1] are shown by the thick lines.

Boxplots of the fitted parameters for the three separate datasets are shown in Fig. 4 along with the parameter values given elsewhere [1] as thicker black lines across the boxplot columns. In many cases, the fitted parameter sets include those found elsewhere within the interquartile range; however, there are also a number of discrepancies. This suggests that there could be correlations between subsets of parameters, all of which give the same output product concentrations, or that the optimisation method has reached local minima with similar score values and root-mean-square errors and not the global minimum (if one exists). We could be more confident that our parameter values are at global minima, if we sampled from more of the parameter space but, with 23 undetermined parameters, if we placed at least one point per edge of the hypercube, this would require more than 96,468,992 parameter sets to try, which is computationally infeasible.

### 7.2 Confidence intervals and correlation coefficients

**Fig. 5:**
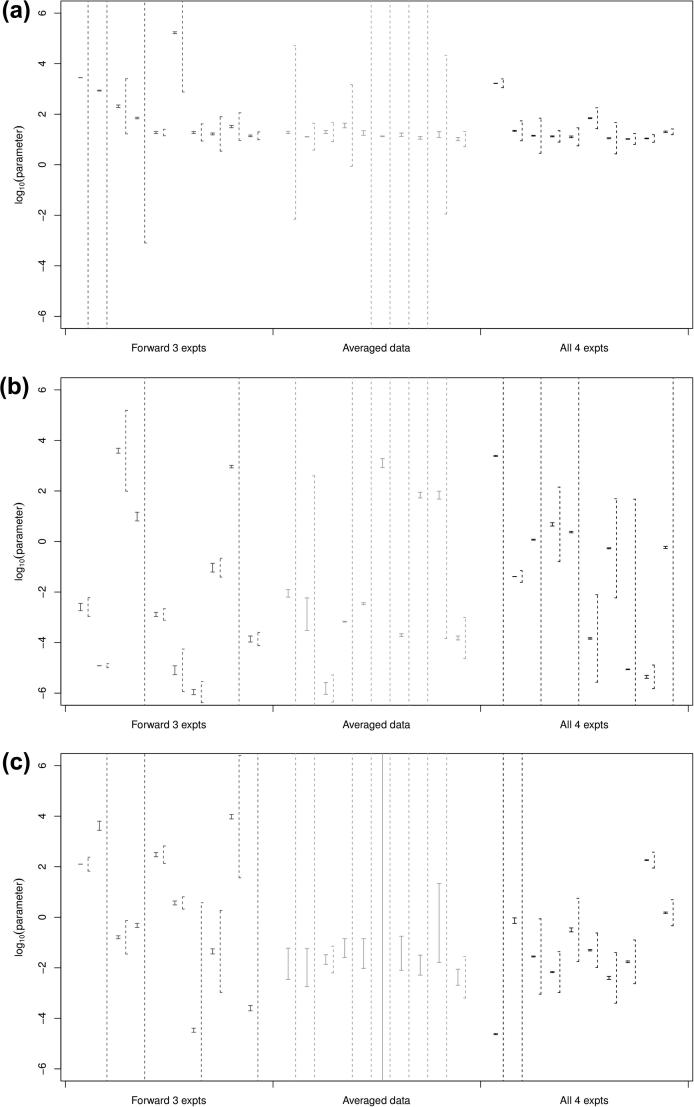
Plot of dependent (solid lines) and independent (dashed lines) confidence intervals for the ten best-scoring parameter sets from: (left) all three forward experiments, (centre) averaged data set, (right) all four experiments (3 forward, 1 reverse): (a) parameter $V_{1}$; (b) parameter $K_{2}$ and (c) parameter $\alpha_{6}$.

**Fig. 6:**
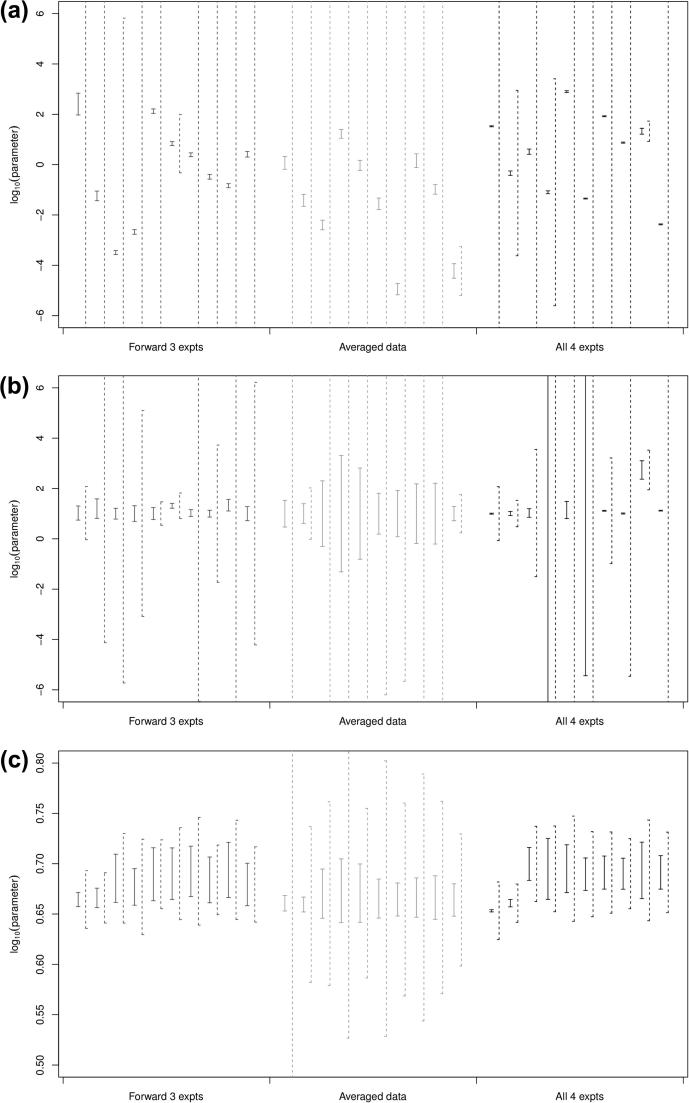
Plot of dependent (solid lines) and independent (dashed lines) confidence intervals for the ten best-scoring parameter sets from: (left) all three forward experiments, (centre) averaged data set, (right) all four experiments (3 forward, 1 reverse): (a) parameter $r_{\mathrm{Ad}}^{+}$; (b) parameter *n* and (c) parameter $p^{*}$.

The dependent and independent 95% confidence intervals for six representative parameters are shown in Figs. 5 and 6 (the parameters $V_{1}\text{,}K_{2}\text{,}\alpha_{6}\text{,}r_{\mathrm{Ad}}^{+}\text{,}n$ and $p^{*}$) (the others are given in the Supplementary material). Note that in all cases, the dependent confidence interval sits inside the independent confidence interval, and that there is a large range of intervals for each parameter. This can only be expected: as the fitted parameters have a large range already (see Fig. 4), the intervals which are necessarily centred on the individual predictions will have a larger range.

Note that in some cases, the independent confidence intervals are very large, with the minimum and maximum outside the [-6,6] interval which we originally used to bound our parameter sets: this, along with the wide variation in the confidence intervals, indicates that the assumptions behind the post hoc fitting of confidence intervals may not hold. Nevertheless, some general trends do appear. The maximum reaction rates $V_{i}$ have small dependent intervals and larger independent intervals, as do the half-saturations $K_{2}\text{,}\;K_{4}\text{,}\;K_{8}$ and $K_{10}$. $K_{1}$ has larger intervals, so is not determined. The factors $\alpha_{3}$ and $\alpha_{6}$ also show the same pattern but, even though $\alpha_{5}\text{,}\;\alpha_{7}$ and $\alpha_{9}$ have small dependent confidence intervals, the independent confidence intervals are much larger than the other two alpha factors, which indicate that these parameters may have a high correlation with parameters which are not well determined. The enzyme reaction rates $r_{x}$ and $r_{x}^{+}$ (for any of the three enzymes) have much larger confidence intervals, which shows that these parameters are not well determined: this is because they have to be inferred from the measured reaction rates using parameters which are themselves uncertain. Most of the choices of *n* and $p^{*}$ within the intervals found lead to the switch function F(p) rapidly switching from 0 to 1 over the pH range, so their exact values are not important.

**Fig. 7:**
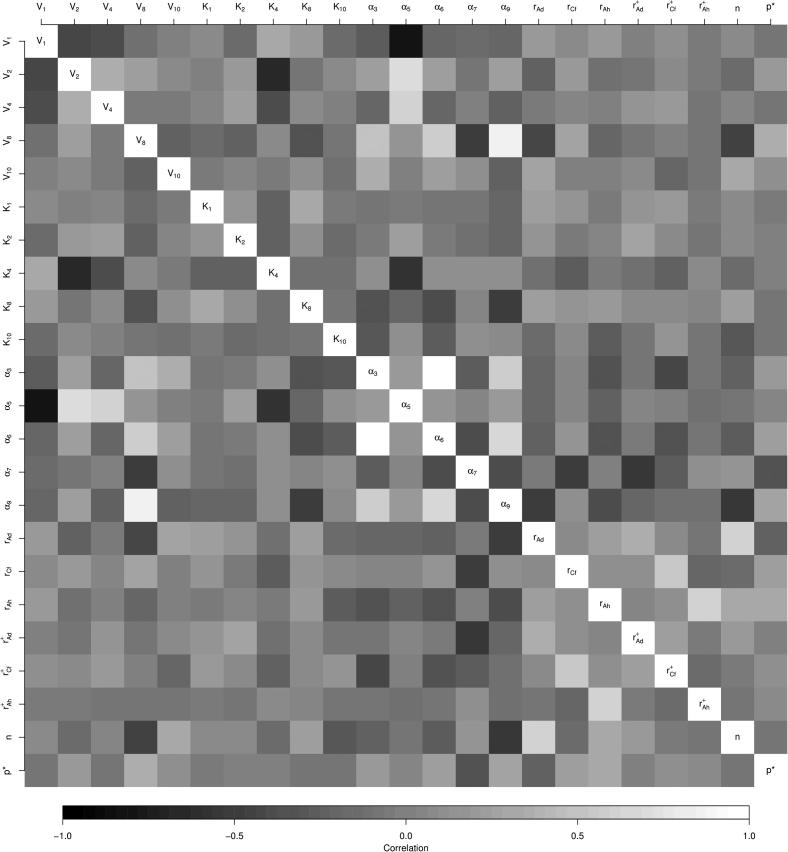
Averaged correlation matrix between the parameters of the lowest 25 scoring parameter sets, averaged over all three ways of organising the data.

The average (over the three parameter sets) of the average correlation coefficients of the 25 lowest-scoring parameter values is shown in Fig. 7 (the averaged correlation matrices split by data grouping are in the Supplementary material). Generally, the signs of the correlations are related to the signs of the relevant reaction terms in the ODE model (for instance, $V_{1}$ and $V_{2}$ are negatively correlated, reflecting that $R_{1}$ and $R_{2}$ have opposite signs in equation for $\frac{\text{d}\mathrm{AC}}{\text{d}t}$ in the model). However, there are exceptions, such as the parameter pair $V_{8}$ and $\alpha_{7}$, which are negatively correlated, but there is no linking ODE. From the metabolic diagram (Fig. 1), the two reactions $R_{8}$ (butyryl-CoA to butyrate) and $R_{7}$ (acetoacetate to acetone) are linked through the reaction $R_{6}$ (butyrate and acetoacetyl-CoA to butyryl-CoA and acetoacetate), and we can see from the correlation diagram that $\alpha_{6}$ and $\alpha_{7}$ are negatively correlated, and $\alpha_{6}$ and $V_{8}$ are positively correlated, so the correlation between $V_{8}$ and $\alpha_{7}$ will be negative. There are two main groups of significantly correlated parameters: those involved in the rate equation for acetyl-CoA (AC), such as $V_{1}\text{,}\;V_{2}\text{,}\;V_{4}\text{,}\;K_{4}$, and $\alpha_{5}$, and those involved in the consumption of acetoacetyl-CoA (AaC), $\alpha_{3}\text{,}\;\alpha_{6}\text{,}\;\alpha_{9}\text{,}\;r_{\mathrm{Cf}}^{+}$, and $\alpha_{7}$: the coefficients $\alpha_{3}$ and $\alpha_{6}$ have an estimated correlation coefficient *r* of 0.98, which is unsurprising as the two reactions $R_{3}$ and $R_{6}$ are mediated by the same group of enzymes (CoA-transferase subunits A and B). What these clusters of correlations indicate is that presently there is redundancy in the model as parameters can vary in defined relationships with each other. To break this redundancy, more data would be needed, particularly the concentrations of the important intermediates acetyl-CoA (AC) and butyryl-CoA (BC), which are branch-points in the metabolism (Fig. 1).

Of course, it may be that the score function does not have ellipsoidal contours around the minima (so the assumption that the likelihood function is proportional to a multivariate normal with mode at the minima may not hold) and the minima themselves may only be local not global.

## 8 Extending the model

It is known that the build up of acids and the change in the external pH triggers spore formation as well as solvent production [2]. However, the exact details are not clearcut [13]. To account for potential spore formation, we split the cells in the culture into two phenotypes, vegetative cells and sporulated cells. The first category will include those cells which have begun the sporulation process, but have not yet reached full sporulation. If we assume that the spores do not metabolise, then the metabolic model (Section 3) becomes:

(8.1) $$\begin{array}{cc} & \frac{\text{d}\mathrm{AC}}{\text{d}t}=(1-\phi )\left(R_{1}-R_{2}+R_{3}-2R_{4}-R_{5}\right)-D\cdot \mathrm{AC}\text{,}\\ & \frac{\text{d}A}{\text{d}t}=(1-\phi )\left(R_{2}-R_{3}\right)-D\cdot A\text{,}\\ & \frac{\text{d}\mathrm{En}}{\text{d}t}=(1-\phi )R_{5}-D\cdot \mathrm{En}\text{,}\\ & \frac{\text{d}\mathrm{AaC}}{\text{d}t}=(1-\phi )\left(R_{4}-R_{3}-R_{6}-R_{10}\right)-D\cdot \mathrm{AaC}\text{,}\\ & \frac{\text{d}\mathrm{Aa}}{\text{d}t}=(1-\phi )\left(R_{3}+R_{6}-R_{7}\right)-D\cdot \mathrm{Aa}\text{,}\\ & \frac{\text{d}\mathrm{BC}}{\text{d}t}=(1-\phi )\left(R_{10}-R_{8}+R_{6}-R_{9}\right)-D\cdot \mathrm{BC}\text{,}\\ & \frac{\text{d}B}{\text{d}t}=(1-\phi )\left(R_{8}-R_{6}\right)-D\cdot B\text{,}\\ & \frac{\text{d}\mathrm{An}}{\text{d}t}=(1-\phi )R_{7}-D\cdot \mathrm{An}\text{,}\\ & \frac{\text{d}\mathrm{Bn}}{\text{d}t}=(1-\phi )R_{9}-D\cdot \mathrm{Bn}\text{.} \end{array}$$

where ϕ(t) is the spore fraction, and the reaction rates $R_{1}\text{,}\ldots \text{,}R_{10}$ are given as previously in Section 3. The internal enzyme concentration differential equations do not change. Note that in the case where ϕ(t)≡0 this reduces to the previous model. For the function ϕ(t), there are a number of choices which could be used, including the following basic models:

• (Model 1a) A step function with fixed height, width and time-at-half-saturation:
  (8.2) $$\phi (t)=\frac{\phi_{\max}}{2}\left(1+\tanh\left(m\left(t-c_{2}-\delta t\right)\right)\right)\text{.}$$
  Restrictions are placed on the parameters $\phi_{\max}\in [0\text{,}0.1]\text{;}\;m\in [10\text{,}1000]\text{;}\;\delta t\in [0\text{,}100]$; where $c_{2}$ is the time of the switch in pH in the particular experiment.
• A production model of the form:
  (8.3) $$\frac{\text{d}\phi }{\text{d}t}=k\left(1-\phi (t)\right)f(t)-D\phi \text{,}$$
  where f(t) is a function which increases from 0 to 1 through the pH transition. The maximum production rate *k* is restricted so that the maximum concentration of spore cells is the same as in the previous non-dynamic model. Three choices for the function f(t) will be adopted:
  – (Model 1b) Constant production rate post $t=c_{2}+\delta t$ via a step-function (with δt>0):
    (8.4) $$f(t)=\frac{1}{2}\left(1+\tanh\left(m\left(t-c_{2}-\delta t\right)\right)\right)\text{.}$$
    This could model a simple threshold switch on the level of the build-up of an as-yet-undetermined protein which is produced once the pH is shifted. *m* is restricted to lie in the interval [10, 1000] and δt∈[0,100].
  – (Model 1c) Production related to pH through F(p), the function relating increased enzyme to the pH.
    (8.5) f(t)=F(p).
    This could model a direct sporulation response to the changes in pH. It assumes that the biochemical processes within the vegetative cell triggering the change in metabolism also trigger the sporulation response.
  – (Model 1d) Production related to pH through F(p(t-δt)) with a delay δt>0:
    (8.6) f(t)=F(p(t-δt)).
    This could model the build-up of pH-activated enzymes inside the cell, triggering sporulation once a threshold is reached.

**Fig. 8:**
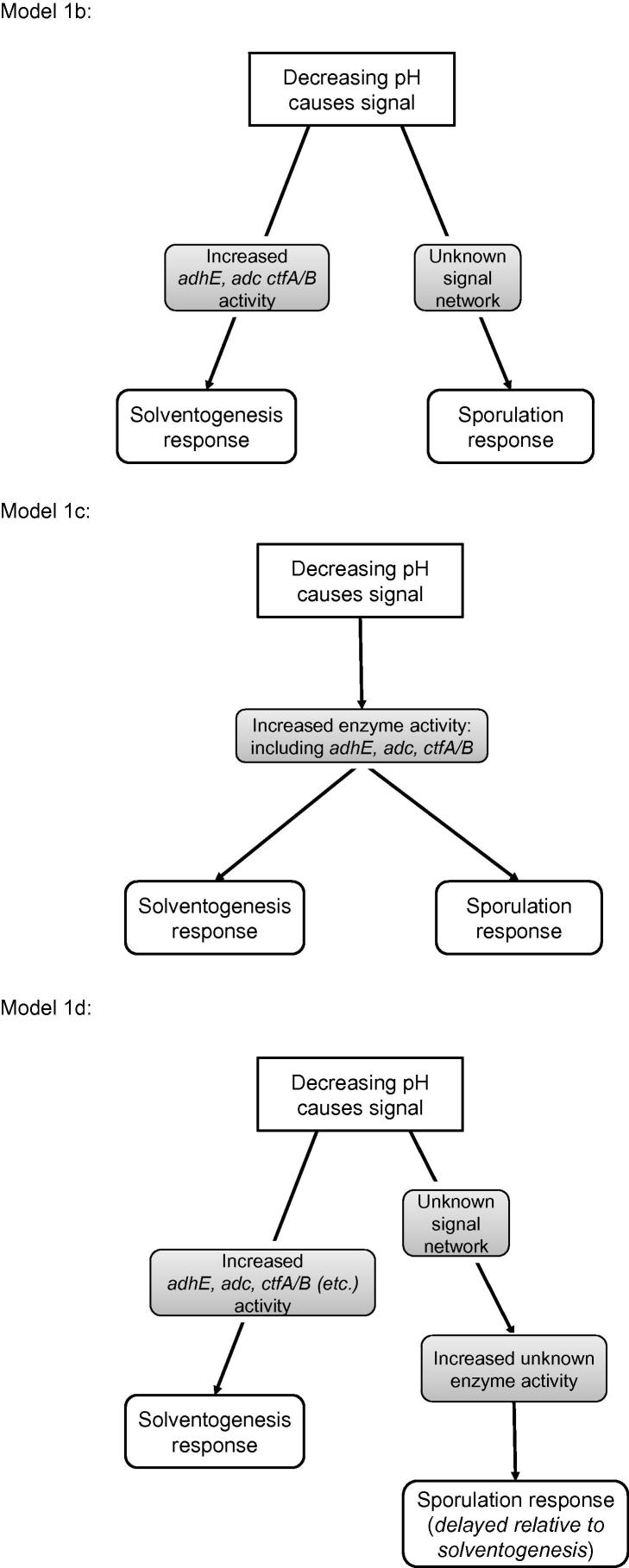
Diagrams of the production models explored in this paper: model 1b, where sporulation and solventogenesis are independently triggered following the pH switch; model 1c, where sporulation and solventogenesis are triggered simultaneously following the pH switch; model 1d, where solventogenesis is triggered following the pH switch, and sporulation occurs after a delay.

All three of the ODE models (i.e. 1b–1d) have the same basic form, namely that the production is stepped up some time after the pH is shifted—however, they differ in their biological interpretation: model 1b assumes that sporulation and solventogenesis are triggered independently of each other through different enzyme pathways; models 1c and 1d assume that the same (presently unknown) enzyme is involved in triggering the solventogenesis and sporulation cascade, both with (1d) and without (1c) a delayed response in sporulation compared to the solventogenesis response (see Fig. 8). The restrictions on *k* are calculated such that it does not exceed the maximum spore production rate (which is in turn directly related to the largest concentration of the sporulation protein Spo0A) used in other work when examining the sporulation cascade in a batch culture [14], which is $9\times 10^{-3}\;{\text{hr}}^{-1}$; when corrected for converting from batch to continuous culture and for dilution this gives an upper limit on $k_{\max}$ of approximately $8\times 10^{-3}\;{\text{hr}}^{-1}$, which gives a limiting spore fraction $\phi_{\max}$ of approximately 0.1, which we take as our maximum.

Biologically, the final three production models are more plausible, as they relate the production of spores to some internal state of the cell. We could use different trigger pHs in the final two models than for the shift in metabolism, but if, as suspected, changes in the level of the same enzyme begin both the metabolic shift and sporulation response [14], then it is presently parsimonious to tie the production of spores to the change in metabolism. There are other suitable choices of production-type model, but such developments would overcomplicate things further—as the parameters in the model are not directly observed, then we could end up with overfitting in the model and/or indeterminable parameters, as we found previously with the enzyme rates. However, if the biological network for pH-triggered sporulation is uncovered, then this would provide a means of determining the correct functional form for the production term f(t), relating it mechanistically to the levels of internal proteins or enzymes.

## 9 Parameter fitting

The same method as in Section 5 was used to fit all parameters, including the extra parameters for each of the four possible sporulation models. We use all three forward experiments as our optimising dataset (as we do not have any germination model for moving from spores to vegetative cells), and optimise over all parameters, since, if there is a significant non-zero spore fraction, the reaction parameters found previously may not apply post-sporulation. Once the parameters and the residual sum of squares are found for each model, the Akaike information criterion (AIC) was calculated via the following formula [9]:

(9.1) AIC=Nlog(RSS/N)+2m,

where *RSS* is the score function (the residual sum of the squared differences between the model and the data), *N* is the number of points to be fitted and *m* is the number of parameters. As the optimisation method is stochastic, we take the lowest 25 scorers (by *RSS* value) and compute the average AIC for the no sporulation model (Section 3) and for the four sporulation models (Section 8). Following standard model selection procedures, the lowest average AIC value is the model to select: the difference in the AICs can be used to estimate the relative weights of evidence from the data behind all five models (including the non-sporulation model) [9].

## 10 Sporulation model results

**Fig. 9:**
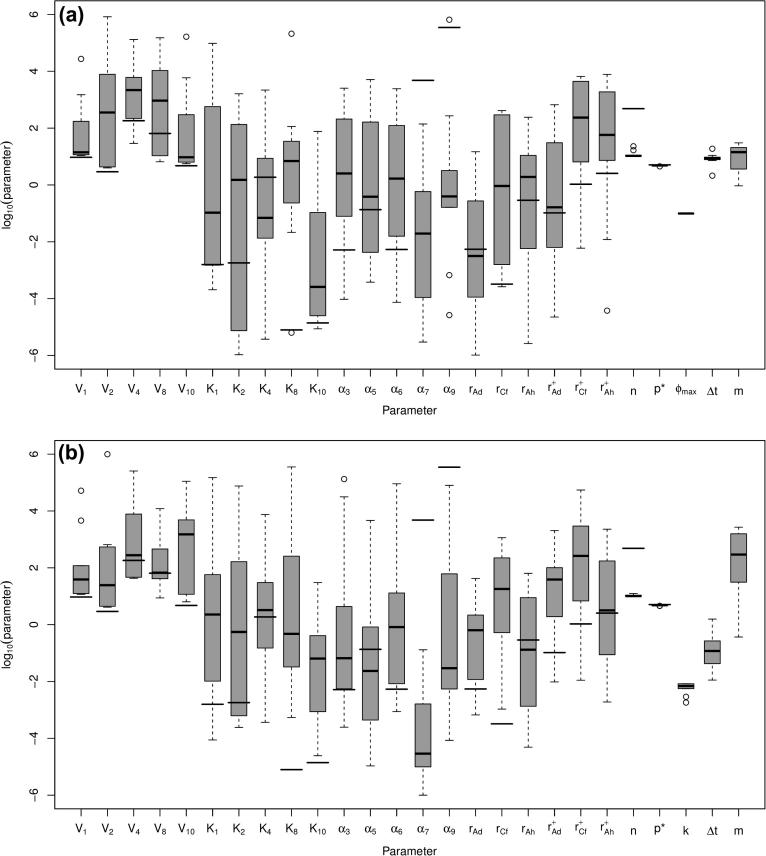
Boxplot of $\log_{10}(\text{parameters})$ for parameters fitted to three forward experiments for sporulation models: (a) Model 1a and (b) Model 1b. Parameters for the non-sporulation model, fitted elsewhere [1] are shown by thick lines.

**Fig. 10:**
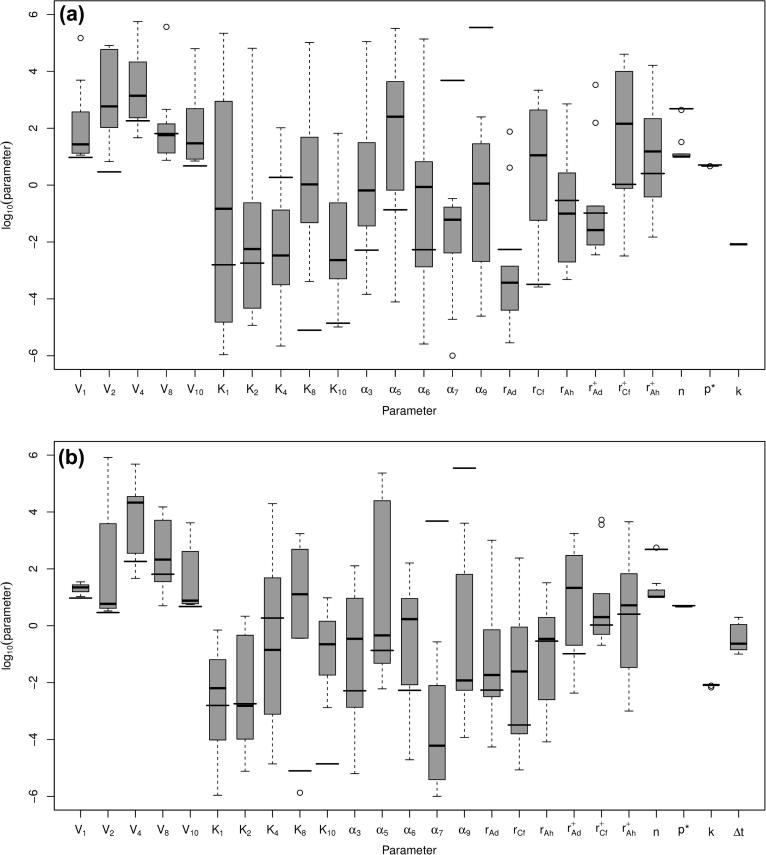
Boxplot of $\log_{10}(\text{parameters})$ for parameters fitted to three forward experiments for sporulation models: (a) Model 1c and (b) Model 1d. Parameters for the non-sporulation model, fitted elsewhere [1] are shown by thick lines.

**Fig. 11:**
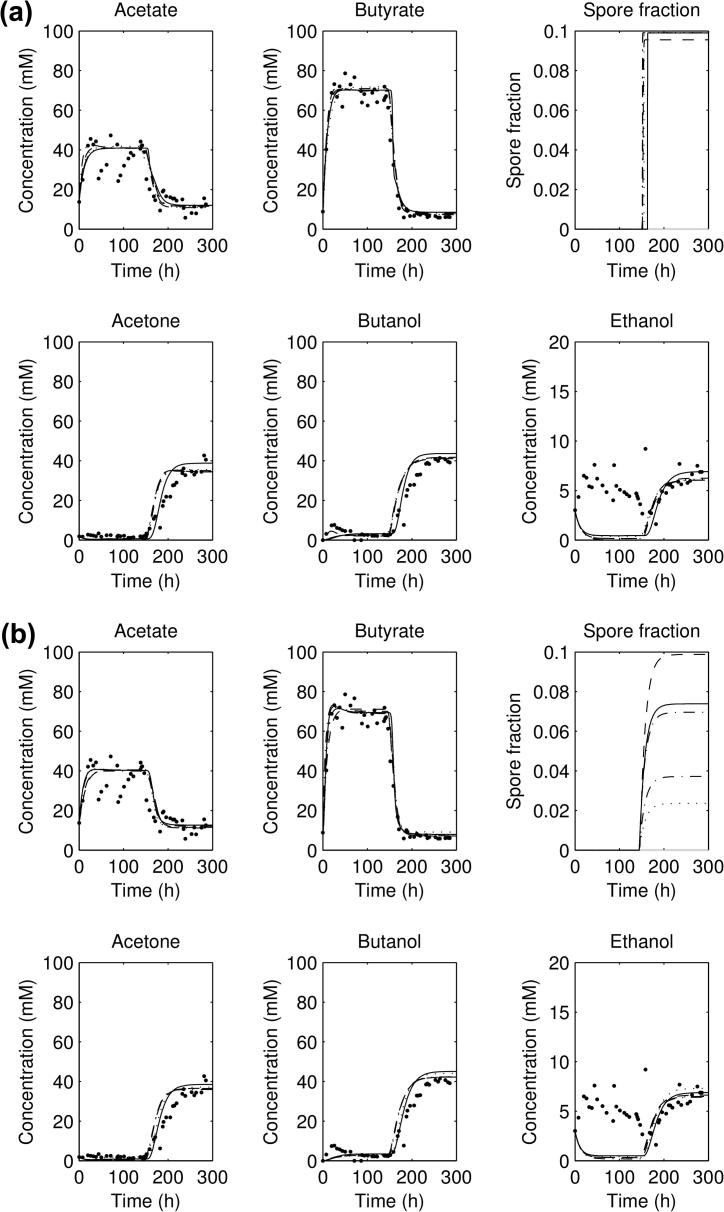
Plot of the acid and solvent concentrations and sporulation fraction for the second forward experiment, using the five lowest-scoring parameter sets for: (a) sporulation model 1a and (b) sporulation model 1b; as continuous lines, with experimental data as points.

**Fig. 12:**
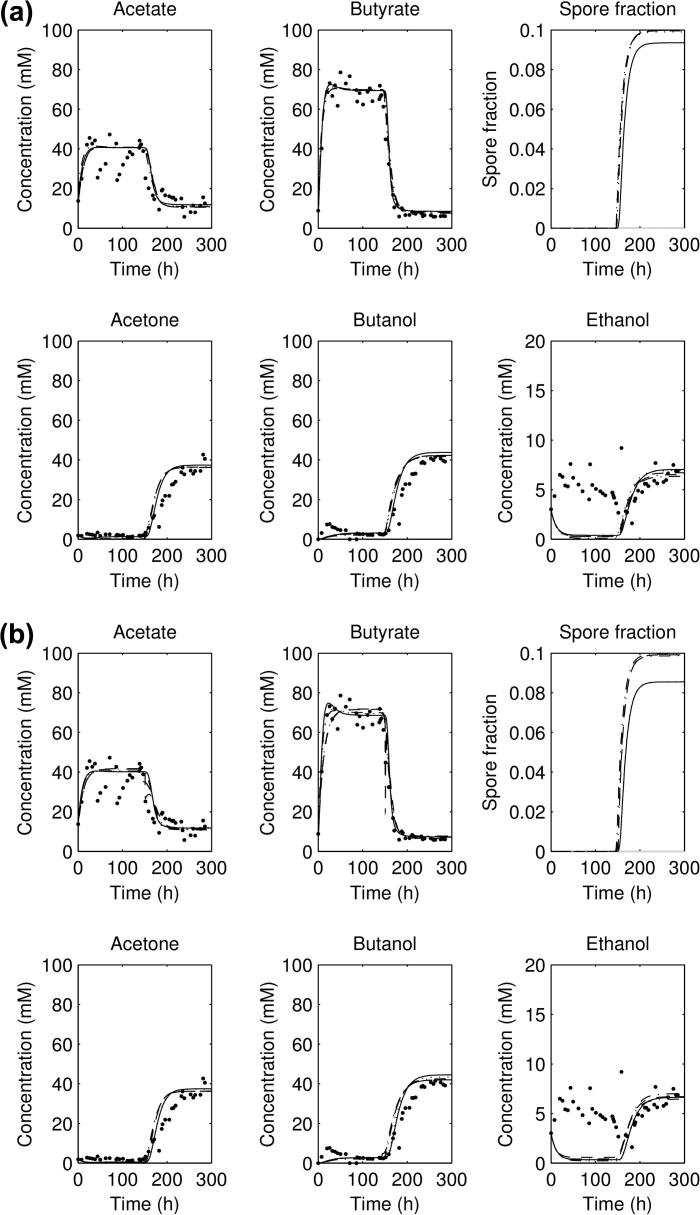
Plot of the acid and solvent concentrations and sporulation fraction for the second forward experiment, using the five lowest-scoring parameter sets for: (a) sporulation model 1c and (b) sporulation model 1d; as continuous lines, with experimental data as points.

**Fig. 13:**
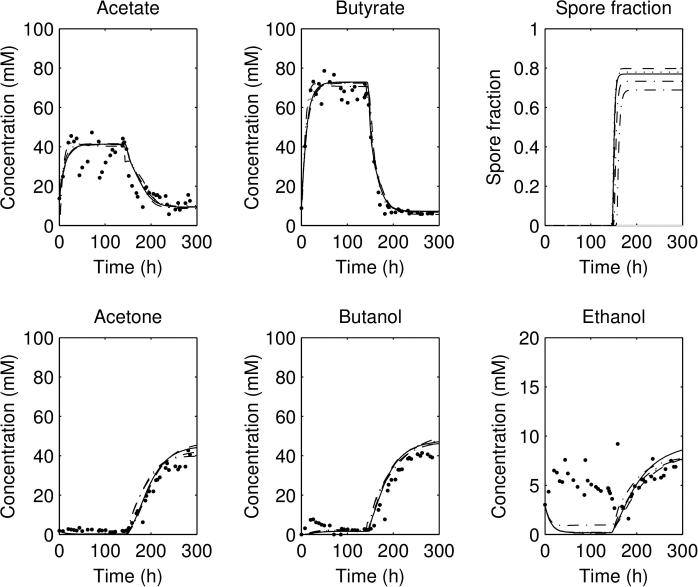
Plot of the acid and solvent concentrations and sporulation fraction for the second forward experiment, using the five lowest-scoring parameter sets with unrestricted spore production rate *k* for sporulation model 1d. Note that the fit for the solvents acetone, butanol and ethanol post-shift are better than in Fig. 12b.

**Fig. 14:**
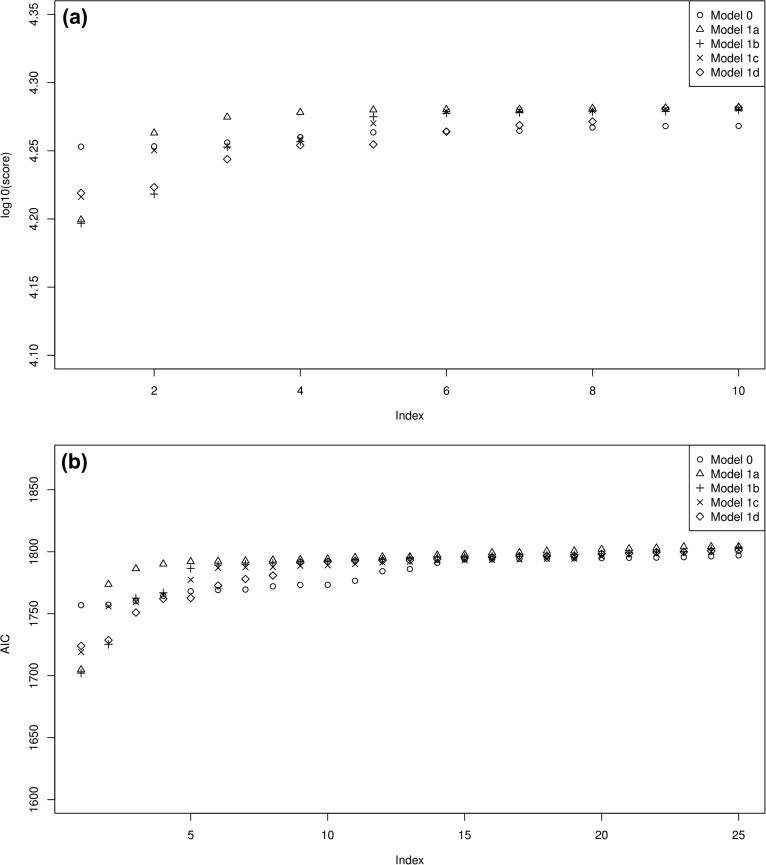
Plot of: (a) $\log_{10}$(residual sum of squares) for the 10 best-fitting parameter sets for the four models: Model 0 (non-sporulation) and the four sporulation models (1a–1b) and (b). Akaike information criterion (AIC) values for the 25 best-fitting parameter sets for the four models: Model 0 (non-sporulation) and the four sporulation models (1a–1b).

The parameter estimates for the models 1a–1d are shown in Figs. 9 and 10: as can be seen, the general spread of the parameters in all of them is not too dissimilar to that for the no sporulation model. However, looking at the results from (lowest five scoring) parameter sets for the four sporulation-included model, all of the four models (Figs. 11 and 12 for the second experimental set: others have the same general behaviour) show a significant sporulation fraction post-pH shift of between 0.03 and 0.1, regardless of the exact model used. Note that the models still underestimate the acid and solvent production in experiments 1 and 3, and overshoot the acid and solvent production in experiment 2. Plots of the lowest 10 scores are shown for the non-sporulation model, and the four sporulation models in Fig. 14b show that including sporulation decreases the residual sum of squares, so to test whether we are overfitting the model, we calculate the AIC for each set of parameters, which can be seen in Fig. 14a. It can be seen that the lowest AICs for the sporulation models are lower than for the non-sporulation one (Table 2): however, the average values over the lowest 25 scoring parameter sets are approximately the same (apart from model 1a). Lower scores are due to the fact that the models appear to fit the post-pH shift data better than the non-sporulation models (and, obviously, ϕ(t) is close to negligible for $t<c_{2}$, so the models are identical before the shift). There is therefore limited evidence that sporulation occurs during the pH shift, but there is comparatively more evidence that sporulation is triggered as part of a cascade post pH-shift. However, if we remove the restriction on the spore formation *k*, this reduces the AIC even further: the lowest AIC value for Model 1d (the lowest scoring of the unrestricted-*k* models; the simulation results are shown in Fig. 13), drops to 1571.6 (with the average over the lowest 25 scorers to 1641.3), which is of the order of 130–160 below the best fits for the restricted *k* (the other sporulation model fits are similar), which would suggest that we would need to accept a model in which more than 75% of the cells do not metabolise post-shift. A large spore fraction such as this would be immediately evident in the culture, but as this is not seen (H. Janssen, personal communication) this suggests that the model as it stands is deficient. Further improvements to this metabolism model, including the introduction of two separate cell populations (one with a primarily acidogenic physiology and one with a primarily solventogenic physiology) may be required to improve the fit further and narrow down the parameter ranges.

## 11 Conclusion

In summary, the parameters we find here (for the no sporulation model), give no better or no worse fit than those found elsewhere [1], which validates the average-interpolated data set approach that they used for fitting their parameters. Simplifications in the original model which are carried over into this model may be responsible for the lack of fit for acid concentrations during the acidogenesis phase, and for the lack of production of ethanol in the simulations during this first stage of the experiments. The range in the parameters (and the size of the confidence intervals around them) is large, which could be due to the parameter fitting method not finding the globally best fit or due to correlations between parameter subsets which give the same output responses. However, we can see that the directly measurable parameters such as the maximum reaction rates, $V_{i}$, and the half-saturations $K_{i}$ in the Michaelis–Menten reaction rates are better determined than the enzyme reaction rates $r_{k}$ and $r_{k}^{+}$: this is because the enzyme rates are inferred from reaction rates and parameters which are already uncertain. The only parameters which are not dependent upon other parameters are the two relating to the first reaction $R_{1}:V_{1}$ and $K_{1}$; this is because this determines the total carbon flux into the cells—as we assume that the glucose concentration in the chemostat is constant, the expression for the reaction rate $R_{1}$ (which should be approximately constant across all realisations) will provide an expression for $K_{1}$ in terms of $V_{1}$ or vice versa. We find that the ranges of these two parameters are smaller than the others in our parameter fits, which is consistent with this.

The signs of the correlations in general are related to the signs of the relevant reaction terms in the ODE model—$V_{1}$ and $V_{2}$ are of opposite signs in the differential equation for *AC*, and are hence negatively correlated. There are some exceptions, where there is no linking ODE, for instance $V_{8}$ and $\alpha_{7}$. The reactions $R_{7}$ (acetoacetate to acetone) and $R_{8}$ (butyryl-CoA to butyrate) are linked through the reaction $R_{6}$ which is the conversion of butyrate and acetoacetyl-CoA and acetoactate and butyryl-CoA: $\alpha_{6}$ is negatively correlated with $\alpha_{7}$, and $\alpha_{6}$ and $V_{8}$ are positively correlated, so we would expect $V_{8}$ and $\alpha_{7}$ to be negatively correlated. We find there are two main groups of significantly correlated parameters: those associated with the rate equation for acetyl-CoA (AC), such as $V_{1}\text{,}\;V_{2}\text{,}\;V_{4}\text{,}\;K_{4}$, and $\alpha_{5}$, and those involved in the metabolism of AaC, $\alpha_{3}\text{,}\;\alpha_{6}\text{,}\;\alpha_{9}\text{,}\;r_{\mathrm{Cf}}^{+}$, and $\alpha_{7}$. The largest correlation between two parameters is that of $\alpha_{3}$ and $\alpha_{6}$ which have an estimated correlation coefficient *r* of 0.98, which is reflective of the fact that the reactions $R_{3}$ and $R_{6}$ are both mediated by CoA-transferase. These correlated clusters indicate that there is either redundancy in the model or that the parameters are underdetermined (a fact borne out by the computed large confidence intervals), for which we would require more metabolite data, particularly those of the branch-point intermediates acetyl-CoA and butyryl-CoA. However, as these two branch-point intermediates are internal rather than external metabolites, their direct concentrations will be difficult to measure: nevertheless, if metabolomic studies are performed which give the concentrations of these internal intermediates relative to the external metabolites during the steady-state solventogenesis and acidogenesis phases and the shift, these will allow the required reaction rates to be narrowed down and the parameters better-determined.

We introduce sporulation into the model, triggered post-shift, which reduces the differences between the model predictions and the data post-shift, but does not reduce the average AIC below that of the no-sporulation model. However, the lowest AIC values are reduced. There is therefore some evidence that sporulation could occur, but more data would be required to confirm this, such as spore fraction assays. The three models which link spore production directly to the pH shift perform better than the simple step-function model—which is consistent with the hypothesis that spore production is linked to the solventogenic response, allowing the organism to survive if conditions worsen significantly. If we allow the spore fraction to reach approximately 75%, we can improve the fit still further (as measured by the Akaike Information Criterion), but a spore fraction of this size was not seen in the experiments. Further improvements to this metabolism model, including the introduction of two separate cell populations (one with a primarily acidogenic physiology and one with a primarily solventogenic physiology) may be required to improve the fit further, but this would require more parameters, and more data to determine them, either through more measurements during the culture or through independent experiments testing specific reactions in the metabolism.

The parameter ranges being large, the large confidence intervals and the clusters of correlated parameters show the network as it stands cannot be determined completely without more data: we suggest that we would need at the minimum the concentrations of the branch point intermediates acetyl-CoA and butyryl-CoA, as well as a spore assay during both phases of the experiment. At present, there is too much leeway in the data to make definitive conclusions about the metabolism, aside from concluding that there could be some weak evidence for sporulation in the data (as the fit is improved by including a non-metabolising fraction). Nevertheless, we are investigating the use of approximate Bayesian methods (such as approximate Bayesian computation via stratified Monte Carlo [15,16]) for generating sensible credibility regions for the parameters in the model and narrow down the parameters further.

## Figures and Tables

**Table 1:** Table showing the parameters estimated in the previous work [1].

Parameter	Units	Value
$V_{1}$	h^−1^	9.41^a^
$V_{2}$	h^−1^	2.92
$V_{4}$	h^−1^	182^a^
$V_{8}$	h^−1^	64.8
$V_{10}$	h^−1^	4.75
$K_{1}$	mM	0.00158
$K_{2}$	mM	0.00181
$K_{4}$	mM	1.87
$K_{8}$	mM	$7.92\times 10^{-6}$
$K_{10}$	mM	$1.40\times 10^{-5}$
$\alpha_{3}$	mM^−2^ h^−1^	0.00517
$\alpha_{5}$	mM^−1^ h^−1^	0.135^a^
$\alpha_{6}$	mM^−2^ h^−1^	0.00537
$\alpha_{7}$	mM^−1^ h^−1^	4790
$\alpha_{9}$	mM^−1^ h^−1^	347000
$r_{\mathrm{Ad}}$	mM h^−1^	0.00547
$r_{\mathrm{Cf}}$	mM h^−1^	0.000324
$r_{\mathrm{Ah}}$	mM h^−1^	0.289
$r_{\mathrm{Ad}}^{+}$	mM h^−1^	0.1037
$r_{\mathrm{Cf}}^{+}$	mM h^−1^	1.063
$r_{\mathrm{Ah}}^{+}$	mM h^−1^	2.5594
*n*	pH^−1^	485
$p^{*}$	pH	5.1^a^
*D*	h^−1^	0.075

a The updated parameters for the corrected model.

**Table 2:** Table showing the highest, lowest and average AIC values (from the lowest 25 scoring parameter sets) for the five models in this paper: model 0 (no sporulation model), 1a–1d (sporulation models).

Model number	Lowest AIC value	Highest AIC value	Average AIC value
0 (no sporulation)	1756.9	1797.0	1781.9
1a	1704.8	1804.2	1792.7
1b	1702.0	1803.8	1786.4
1c	1719.0	1801.6	1785.8
1d	1724.1	1801.1	1783.8
