# Supplementary material for: pH-induced gene regulation of solvent production by Clostridium acetobutylicum in continuous culture: Parameter estimation and sporulation modelling
Source: Math Biosci. 2013 Feb;241(2):149–66. doi: 10.1016/j.mbs.2012.11.004 (PMC3547174; doi:10.1016/j.mbs.2012.11.004)
Supplement: Supplementary file 1 [file mmc2.pdf]

Supplementary material for “pH-induced gene regulation on solvent production by *Clostridium acetobutylicum* in continuous culture: parameter estimation and sporulation modelling”: optimisation method

G. J. Thorn, J. R. King and S. Jabbari

December 6, 2012

The details of the modified simplex method used for parameter fitting runs as follows (in this and the following, $\mathbf{x}$ are the scaled, log-transformed parameter values).

1. Choose an initial feasible starting point with all m parameters in the range $[0, 1]$ and compute the score function for this point
$$S(\mathbf{x}_0) = \sum_{\text{all data points}} (y(\mathbf{x_0}, t_i) - \tilde{y}_i)^2$$
where $y(\cdots)$ is the output of solving the ODE system (using the stiff ODE solver MATLAB routine `ode15s`) for the continuous culture model or the sporulation-inclusive model for all experiments considered, and $\tilde{y}$ is the particular experimental dataset. If the integration fails, for instance if the minimum step size required for the right solution accuracy is too small, continue to choose a different starting point until the integration is successful; at other times, set the score to a very high value—this ensures that there is at least one feasible point in the simplex at all times.

2. Calculate an initial temperature [1] $T_0 = S(\mathbf{x_0}) \times 10^5$.

3. To generate m other points of the simplex, sample a new point x'_i from [0,1], and create the vectors $\mathbf{x}_i = (x_1, \ldots, x_{i-1}, x'_i, x_{i+1}, \ldots, x_m)$ for $i = 1, \ldots, m$.

4. Compute the score for each point in the simplex. Set the unsuccessful move counter UM, the successful move counter SM, and the unsuccessful costs accumulator UC to zero.

5. For a fixed number of iterations at each temperature do the following.

   - Add a logarithmically distributed random variable to the score at each point
   $$S^*(\mathbf{x}_n) = S(\mathbf{x}_n) + |\log(X)| \cdot T$$
   where X is uniformly distributed on $[0, 1]$.
   - Reorder the points in the simplex with the lowest modified score first $\{\hat{\mathbf{x}}_0, \ldots, \hat{\mathbf{x}}_m\}$.
   - As in the Nelder-Mead simplex algorithm, take the point with the largest modified score, and reflect through the hyperplane containing all of the other points:
   $$\mathbf{x}^* = \hat{\mathbf{x}}_m - 2\left(\hat{\mathbf{x}}_m - \frac{1}{m}\sum_{i=0}^{m-1} \hat{\mathbf{x}}_i\right).$$
   - Compute a modified score for the new point
   $$S_*(\mathbf{x}^*) = S(\mathbf{x}^*) - |\log(X)| \cdot T$$
   where X is uniformly distributed on $[0, 1]$.
   - If the modified score for the new point is less than $S^*(\hat{\mathbf{x}}_0)$, then:

– extrapolate the new point by a factor 2 from the hyperplane containing the other points

$$\mathbf{x}^{**} = \hat{\mathbf{x}}_m - 3\left(\hat{\mathbf{x}}_m - \frac{1}{m}\sum_{i=0}^{m-1}\hat{\mathbf{x}}_i\right);$$

– compute a modified score for the new point

$$S_*(\mathbf{x}^{**}) = S(\mathbf{x}^{**}) - |\log(X)| \cdot T$$

where X is uniformly distributed on $[0, 1]$;

– if the modified score for the new point is lower than that for $\mathbf{x}^*$ then accept the new point $\mathbf{x}^{**}$ and discard the old highest scoring point $\hat{\mathbf{x}}_m$;

– if the modified score for the new point is not, then accept $\mathbf{x}^*$ as the new point and discard $\hat{\mathbf{x}}_m$.

- If the modified score for the new point is at least $S^*(\hat{\mathbf{x}}_m)$, then:

– contract the new point by a factor 1/2 towards the hyperplane containing the other points

$$\mathbf{x}^{**} = \frac{1}{2}\left(\mathbf{x}^* + \frac{1}{m}\sum_{i=0}^{m-1}\hat{\mathbf{x}}_i\right);$$

– compute a modified score for the new point

$$S_*(\mathbf{x}^{**}) = S(\mathbf{x}^{**}) - |\log(X)| \cdot T$$

where X is uniformly distributed on $[0, 1]$;

– if the modified score for the new point is lower than that for $\mathbf{x}^*$ then accept the new point $\mathbf{x}^{**}$ and discard $\hat{\mathbf{x}}_m$.

– if the modified score for the new point is not, then contract all points $\mathbf{x}_i$, $i = 1 \ldots m$, towards $\mathbf{x}_0$:

$$\mathbf{x}_i = \frac{1}{2}\left(\mathbf{x}_0 + \mathbf{x}_i\right).$$

- If the modified score for the new point is less than $S^*(\hat{\mathbf{x}}_{m-1})$, the second highest then accept the new point, and discard $\hat{\mathbf{x}}_m$.
- If the (unmodified) score of the new point is lower than $S(\hat{\mathbf{x}}_0)$ then consider the move successful ($SM := SM + 1$); otherwise, consider the move unsuccessful ($UM := UM + 1$), and add the difference in score between the lowest point and the new point (or the highest scoring point in the contracting case) to the unsuccessful costs accumulator ($UC := UC + S(\text{new point}) - S(\hat{\mathbf{x}}_0)$).

6. The next temperature T_{next} is computed in one of two ways:

- For the first step, the next temperature is calculated as follows:

$$T_{next} = -\left(\frac{UC}{UM + SM}\right) \cdot \frac{1}{\log\left(\frac{0.95\cdot(UM+SM)-SM}{UM}\right)}$$

using the successful and unsuccessful move counters (SM, UM) and the unsuccessful cost accumulator UC.

- For second and subsequent steps, first calculate the standard deviation of the scores of the vertices of the final simplex σ_y then calculate the next temperature by:

$$T_{next} = T \cdot \frac{1}{1 + T\left(\log\left(\frac{1+CR}{3\sigma_y}\right)\right)},$$

where CR is the cooling rate, which was set at 10 for this optimisation (lower CRs give slower convergence, higher CRs give faster convergence but are more likely to hit local rather than global minima [1]).

7. Return to step 3 using the point with the lowest score as the initial point $\mathbf{x}_0$. If $T_{next} < 1$, then return to step 3 with $T_{next} = 0$, where the inner loop reduces to a version of the Nelder-Mead simplex algorithm.

This variation of the simplex algorithm with temperature variation allows the inner loop to take downhill steps most of the time (where the score decreases), whilst occasionally taking an uphill step with a probability which reduces as the temperature T falls. This ensures that the algorithm behaves similarly to a Metropolis-type simulated annealing algorithm for large T, and to a direct Nelder-Mead simplex when T is zero. In our case, when parameter fitting, we ran the above algorithm followed by a local simplex search (the above algorithm with $T = 0$) to give our parameter estimates.

References

[1] M. Cardoso, R. Salcedo, and S. Feyo de Azevedo. The simplex-simulated annealing approach to continuous non-linear optimization. *Computers & Chemical Engineering*, 20(9), 1996.
