# Supplementary material for: pH-induced gene regulation of solvent production by Clostridium acetobutylicum in continuous culture: Parameter estimation and sporulation modelling
Source: Math Biosci. 2013 Feb;241(2):149–66. doi: 10.1016/j.mbs.2012.11.004 (PMC3547174; doi:10.1016/j.mbs.2012.11.004)
Supplement: Supplementary file 2 [file mmc1.pdf]

Supplementary material for "pH-induced gene regulation on solvent production by *Clostridium acetobutylicum* in continuous culture: parameter estimation and sporulation modelling": experimental data in graphical and tabular form

G. J. Thorn, J. R. King and S. Jabbari

December 6, 2012

Four pH-shift continuous culture experiments were performed, three with a forward shift from pH 5.7 to 4.5, and one reverse shift from 4.5 to 5.7:

- 'Forward 1': acidogenesis was maintained for 137 hours, then the pH control was stopped, allowing the metabolic shift to occur. 22 hours after the removal of the pH control the shift had completed, and final measurement was taken at 215 hours.
- 'Forward 2': as in 'Forward 1'—the pH control was switched off after 137.5 hours, the metabolic shift occurred over 33.5 hours and steady state was reached at approximately 236 hours.
- 'Forward 3': as in the previous two, but the pH control was switched off at 121 hours, the metabolic shift lasted 29 hours and the final measurement was taken at 215 hours.
- 'Reverse': the pH was controlled via the addition of KOH so that the change was in the opposite direction—the pH was initially kept low for 129 hours, and then increased over 17 hours to the high level.

For the four experiments (three forward, one reverse), the data for the concentrations of acetate, butyrate, ethanol, acetone and butanol and the pH levels are shown in figures S-1 and S-2, and also in tables S-1–S-4.

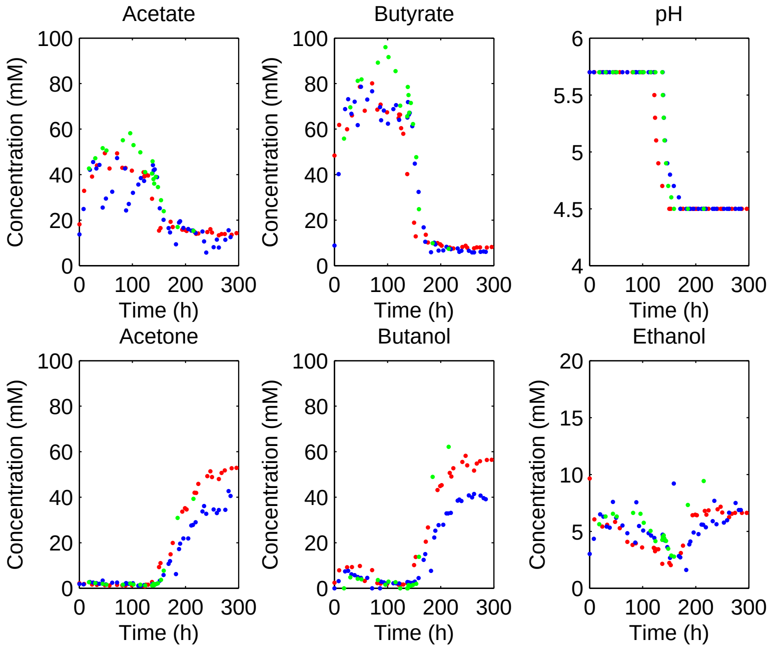

Figure S-1: Plots of the experimental data for the three forward experiments: acetate, butyrate, ethanol, acetone and butanol concentrations, and pH levels: green symbols (‘forward 1’), blue symbols (‘forward 2’), red symbols (‘forward 3’).

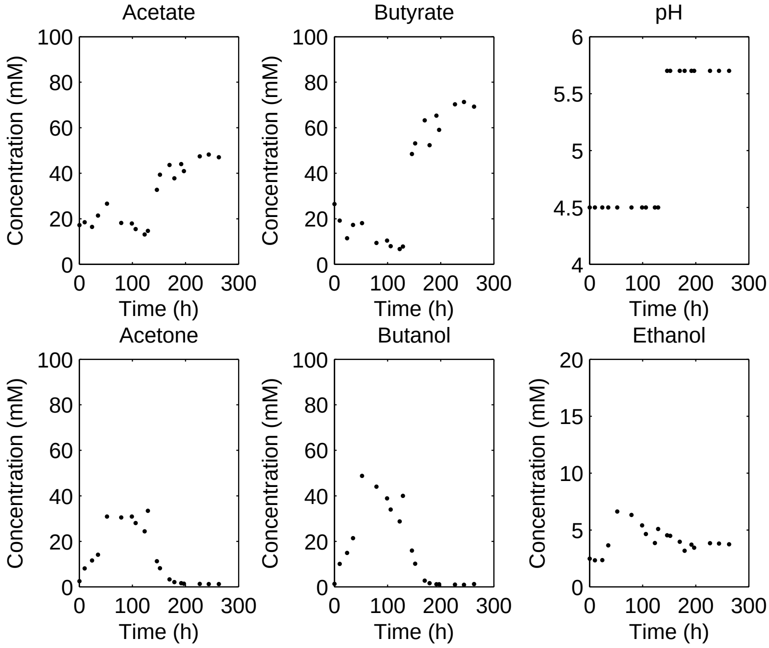

Figure S-2: Plots of the experimental data for the reverse experiment: acetate, butyrate, ethanol, acetone and butanol concentrations, and pH.

Time (hr)	Ethanol (mM)	Acetone (mM)	Acetate (mM)	Butanol (mM)	Butyrate (mM)	pH
18	5.63	2.66	42.65	0	55.83	5.7
30	6.31	2.23	47.2	4.78	69.58	5.7
44	6.55	1.92	51.59	4.12	81.24	5.7
51	6.31	1.58	50.59	3.93	81.84	5.7
82	6.62	1.54	55.14	3.6	89.24	5.7
96	6.55	2.13	58.22	1.55	95.98	5.7
102	5.75	1.59	52.97	2.89	91.67	5.7
115	5.04	1.51	49.81	2.66	85.51	5.7
124	4.13	1.21	41.08	0	70.31	5.7
137	4.25	1.25	40.5	0	65.62	5.7
138	4.51	1.21	45.88	0	78.5	5.5
139.5	4.64	1.95	38.01	1.35	74.99	5.3
141	4.34	1.34	36.06	1.1	67.23	5.1
144	4.16	1.76	39.05	1.05	71.5	4.9
148	3.47	2.16	34.57	1.44	62.19	4.7
154	2.87	3.67	28.72	1.92	47.64	4.6
159	2.77	7.74	23.93	13.79	24.78	4.5
185	7.32	30.89	16.96	48.91	9.87	4.5
215	9.42	39.28	15.31	62.11	7.42	4.5

Table S-1: Table showing the data for the first forward experiment: product concentrations (ethanol, acetone, acetate, butanol, butyrate) and pH levels.

Time (hr)	Ethanol (mM)	Acetone (mM)	Acetate (mM)	Butanol (mM)	Butyrate (mM)	pH
0	3.02	1.82	13.73	0	8.81	5.7
8	4.35	1.7	24.92	3.09	40.26	5.7
20	6.49	2.76	42.15	7.42	68.79	5.7
26	6.29	2.47	45.5	7.63	73.16	5.7
32	5.42	2.26	42.72	6.16	66.71	5.7
38	5.33	1.92	44.27	5.74	72	5.7
44	7.58	3.34	25.55	4.94	61.76	5.7
50	6.2	1.72	29.46	4.52	78.59	5.7
62	5.52	2.42	32.5	4.55	72.95	5.7
71	4.83	2.5	47.25	0	76.6	5.7
86	4.01	1.77	42.68	0	69.64	5.7
88	7.56	2.23	24.22	2.9	63.91	5.7
93	5.46	1.9	27.12	2.58	68.18	5.7
101	5.07	2.21	32.04	2.58	62.42	5.7
111	4.84	1.15	35.68	2.16	68.81	5.7
116	4.63	1.12	38.47	2.19	70.55	5.7
122	4.42	1.39	37.23	2.1	64.07	5.7
137.5	4.7	1.88	40.53	2.74	65.04	5.7
138.5	4.68	1.73	44.18	2.51	71.9	5.5
140	4.4	1.69	41.89	2.55	66.35	5.3
142	4.19	1.7	42.27	2.44	67.15	5.1
146.5	3.62	2.05	38.92	2.37	61.26	4.9
151.5	2.66	3.31	25.2	2.91	44.79	4.8
158.5	9.2	5.83	20.13	4.46	32.4	4.7
168	2.82	10.73	16.52	12.43	16.8	4.6
171	2.71	11.87	14.59	14.99	10.51	4.5
182	1.61	6.2	9.39	7.62	5.88	4.5
188	3.86	17.24	18.93	22.25	10.12	4.5
190	4.11	19.6	19.51	25.25	9.31	4.5
196	4.89	21.88	16.59	27.71	6.61	4.5
205	4.76	21.87	16.12	27.86	6.71	4.5
211	5.6	27.59	15.36	32.88	8.29	4.5
214	5.57	27.84	15.47	32.84	7.69	4.5
219	5.36	28.98	14.34	33.13	7.22	4.5
232	5.9	33.78	14.97	38.5	7.58	4.5
235	7.68	36.08	10.61	38.93	6.06	4.5
239	5.62	32.71	5.77	38.34	6.55	4.5
253	5.77	34.6	8.08	40.77	6.53	4.5
259	5.98	33.06	11.43	39.93	5.78	4.5
263	6.63	34.38	7.98	41.41	5.78	4.5
275	7.49	34.47	11.35	40.71	6.03	4.5
281	6.88	42.68	15.54	39.59	6.2	4.5
285	6.87	40.52	12.55	39.13	6.06	4.5
309	6.06	36.65	10.68	35.1	4.42	4.5

Table S-2: Table showing the data for the second forward experiment: product concentrations (ethanol, acetone, acetate, butanol, butyrate) and pH levels.

Time (hr)	Ethanol (mM)	Acetone (mM)	Acetate (mM)	Butanol (mM)	Butyrate (mM)	pH
0	9.64	2.15	18.15	2.39	48.37	5.7
9	6.05	1.87	32.89	7.9	61.8	5.7
24	5.42	1.66	39.1	9.22	59.93	5.7
33	5.57	1.43	43.99	9.32	66	5.7
48	5.83	1.36	49.44	9.74	78.63	5.7
57	5.27	1.24	42.65	3.19	68.08	5.7
71	4.07	1.49	49.35	7.99	80.12	5.7
81	3.8	1.29	43	2.26	68.59	5.7
87	3.95	1.26	42.95	2	70.79	5.7
99	3.58	1.29	41.7	1.84	67.39	5.7
121	3.55	1.06	41.02	1.58	64.86	5.7
122	3.5	0.99	39.12	1.84	66.38	5.5
123.5	3.25	0.87	39.39	1.66	66.35	5.3
125.5	3.42	1.56	40.12	1.65	60.4	5.1
129.5	3.42	1.56	39.62	1.61	57.95	4.9
137	2.15	2.76	29.37	2.21	40.23	4.7
150	2.23	9.32	15.4	10.2	18.84	4.5
153	2.04	11.01	16.49	13.71	12.85	4.5
172	3.09	14.92	19.28	20.48	13.53	4.5
176	3.75	19.89	16.99	26.73	10.13	4.5
194	6.41	33.65	15.8	43.12	9.95	4.5
199	6.46	35.18	15.73	44.86	9.38	4.5
202	6.42	34.62	15.19	45.29	8.77	4.5
217	6.79	41.98	14.87	50.66	7.97	4.5
220	6.46	41.86	13.97	49.06	7.41	4.5
224	6.85	45.83	14.14	52.72	7.59	4.5
241	6.94	49.25	14.74	55.46	8.15	4.5
247	7.16	51.35	16	58.22	8.77	4.5
250	6.66	48.82	14.48	53.97	7.86	4.5
263	6.25	47.95	13.36	51.72	7.67	4.5
268	6.54	50.65	13.88	54.76	8	4.5
274	6.62	51.8	13.87	55.83	8.01	4.5
287	6.62	52.7	13.6	56.36	7.94	4.5
296	6.62	52.88	14.28	56.43	8.22	4.5

Table S-3: Table showing the data for the third forward experiment: product concentrations (ethanol, acetone, acetate, butanol, butyrate) and pH levels.

Time (hr)	Ethanol (mM)	Acetone (mM)	Acetate (mM)	Butanol (mM)	Butyrate (mM)	pH
0	2.48	2.52	17.23	1.28	26.43	4.5
10	2.34	8.07	18.44	10.12	19.22	4.5
24	2.35	11.62	16.43	14.95	11.45	4.5
35	3.64	14.15	21.38	21.37	17.27	4.5
52	6.62	30.88	26.61	48.74	18.05	4.5
79	6.32	30.46	18.16	43.99	9.35	4.5
99	5.41	30.87	17.92	38.90	10.44	4.5
106	4.64	28.06	15.5	33.94	7.93	4.5
123	3.86	24.38	13.12	28.75	6.71	4.5
129	5.09	33.42	14.64	39.96	7.79	4.5
146	4.54	11.27	32.73	15.93	48.45	5.7
152	4.49	8.19	39.36	10.18	53.13	5.7
170	3.96	3.26	43.64	2.72	63.23	5.7
179	3.17	2.08	37.75	1.59	52.31	5.7
192	3.71	1.62	44.04	1.17	65.3	5.7
197	3.44	1.4	40.96	1.16	59.04	5.7
227	3.84	1.34	47.42	0.95	70.32	5.7
244	3.8	1.25	48.25	0.94	71.36	5.7
263	3.74	1.23	47.02	1.20	69.27	5.7

Table S-4: Table showing the data for the reverse experiment: product concentrations (ethanol, acetone, acetate, butanol, butyrate) and pH levels.
