# Supplementary material for: pH-induced gene regulation of solvent production by Clostridium acetobutylicum in continuous culture: Parameter estimation and sporulation modelling
Source: Math Biosci. 2013 Feb;241(2):149–66. doi: 10.1016/j.mbs.2012.11.004 (PMC3547174; doi:10.1016/j.mbs.2012.11.004)
Supplement: Supplementary file 3 [file mmc3.pdf]

Supplementary material for "pH-induced gene regulation on solvent production by *Clostridium acetobutylicum* in continuous culture: parameter estimation and sporulation modelling": confidence interval and correlation coefficient calculation and results

G. J. Thorn, J. R. King and S. Jabbari

December 6, 2012

S-1 Calculating the correlation coefficients and estimated correlation coefficients

The method for calculating the confidence ellipsoid around the parameters $\hat{\mathbf{x}}$ and the corresponding dependent and independent confidence intervals and correlation coefficients runs as follows (see [1] and references therein):

- Define the discrepancy vector between the model output $y(\mathbf{x}, t_i)$ and the experimental data $\tilde{y}$ for a given parameter vector $\mathbf{x}$:
$$\mathbf{Y}(\mathbf{x}) = y(\mathbf{x}, t_i) - \tilde{y}_i$$
so that the score function can be written as
$$S(\mathbf{x}) = \sum_{\text{all data points}} (y(\mathbf{x}, t_i) - \tilde{y}_i)^2 = \mathbf{Y}(\mathbf{x})^{\mathbf{T}}\mathbf{Y}(\mathbf{x}),$$
then the Jacobian (number of data points $N\times$ number of components m) of the solution can be written as
$$J_{ij}(\mathbf{x}) = \frac{\partial Y_i}{\partial x_j},$$
which is the sensitivity of the ith component of the discrepancy vector to small changes in the parameter x_j.
- Decompose the Jacobian at the minimum $\hat{\mathbf{x}}$ using singular value decomposition
$$J(\hat{\mathbf{x}}) = U(\hat{\mathbf{x}})\Sigma(\hat{\mathbf{x}})V^T(\hat{\mathbf{x}}).$$
Assuming the likelihood is multivariate normal, with the mode at the minimum $\hat{\mathbf{x}}$, we can compute the $1-\alpha$ confidence ellipsoid
$$(\mathbf{x} - \hat{\mathbf{x}})^T(J^TJ)(\mathbf{x} - \hat{\mathbf{x}}) \le \frac{m}{N-m}S(\hat{\mathbf{x}})F_\alpha(m, N-m),$$
where F_α is the upper α point of the F-distribution with m and $N-m$ degrees of freedom.
- Invert the above, noting that $J^TJ = V\Sigma^2V^T$, and let $r_\sigma^2 = \frac{m}{N-m}S(\mathbf{x})F_\alpha(m, N-m)$, so the intervals can be written as follows:
$$|x_i - \hat{x}_i| \le \frac{r_\sigma}{\sqrt{(V\Sigma^2V^T)_{ii}}}$$
for the dependent interval, and
$$|x_i - \hat{x}_i| \le r_\sigma\sqrt{(V\Sigma^{-2}V^T)_{ii}}$$
for the independent interval, with the correlation coefficients between the i and j components of the parameter vector given by
$$\rho_{ij} = \frac{B_{ij}}{\sqrt{B_{ii}B_{jj}}}$$
where
$$B_{ij} = (V\Sigma^2V^T)_{ij}.$$

S-2 Confidence intervals and correlation plots

The confidence intervals for all 23 parameters are shown here in figures S-1–S-23, with the 6 parameters shown in the main text included for comparison purposes.

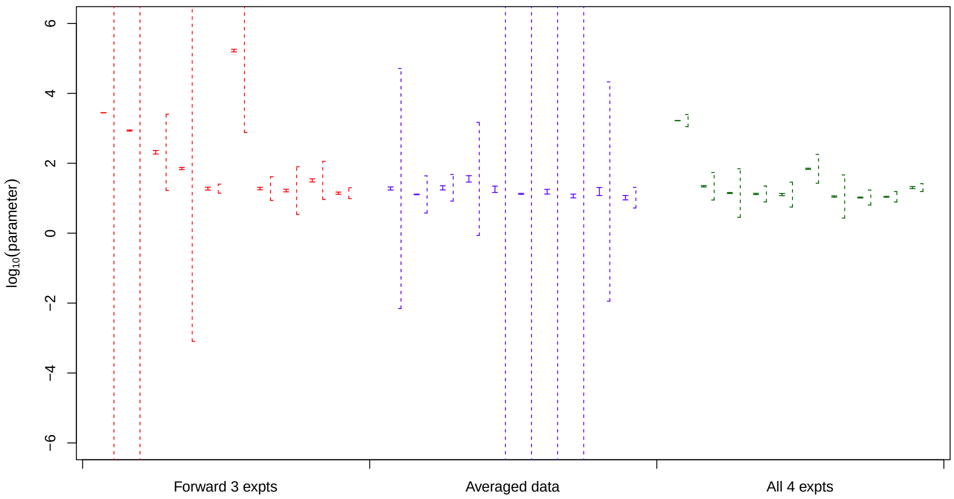

Figure S-1: Plot of dependent (solid lines) and independent (dashed lines) confidence intervals for the parameter V_1, for the ten best-scoring parameter sets from: a) (red) all three forward experiments, b) (blue) averaged data set, c) (green) all four experiments (3 forward, 1 reverse)

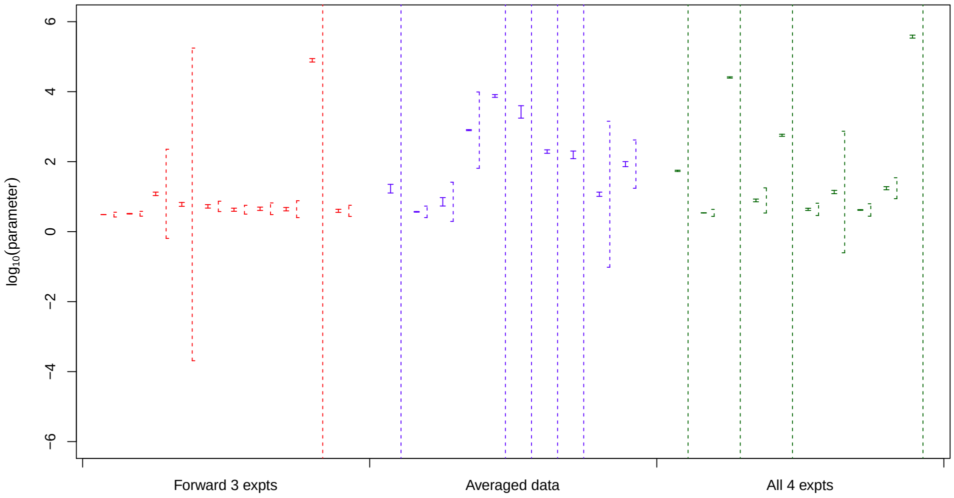

Figure S-2: Plot of dependent (solid lines) and independent (dashed lines) confidence intervals for the parameter V_2, for the ten best-scoring parameter sets from: a) (red) all three forward experiments, b) (blue) averaged data set, c) (green) all four experiments (3 forward, 1 reverse)

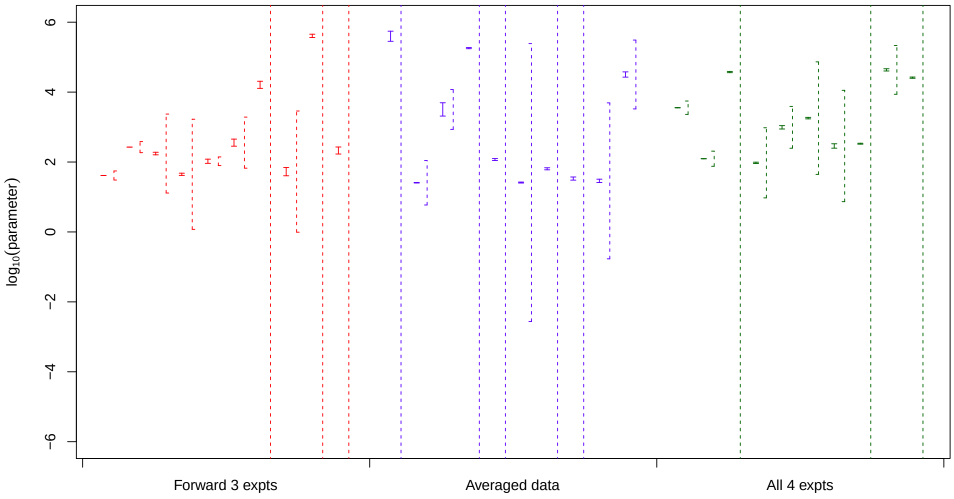

Figure S-3: Plot of dependent (solid lines) and independent (dashed lines) confidence intervals for the parameter V_4, for the ten best-scoring parameter sets from: a) (red) all three forward experiments, b) (blue) averaged data set, c) (green) all four experiments (3 forward, 1 reverse)

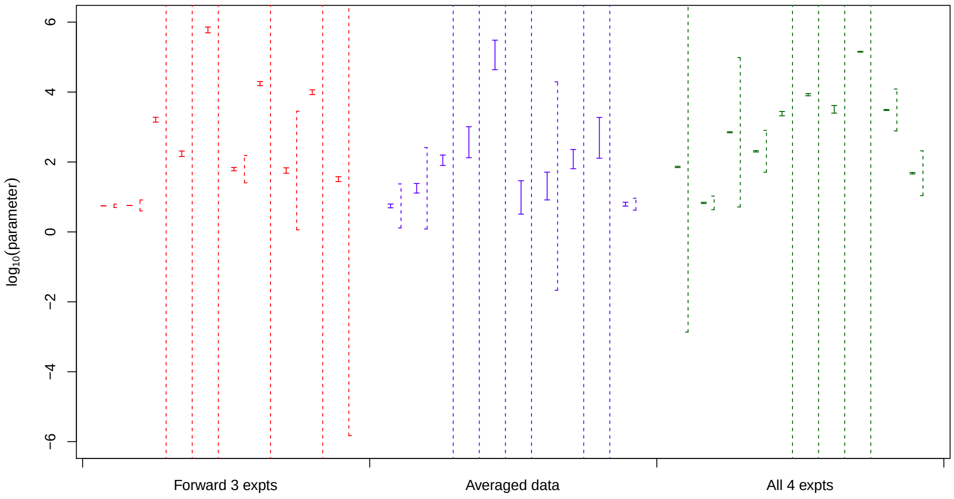

Figure S-4: Plot of dependent (solid lines) and independent (dashed lines) confidence intervals for the parameter V_8, for the ten best-scoring parameter sets from: a) (red) all three forward experiments, b) (blue) averaged data set, c) (green) all four experiments (3 forward, 1 reverse)

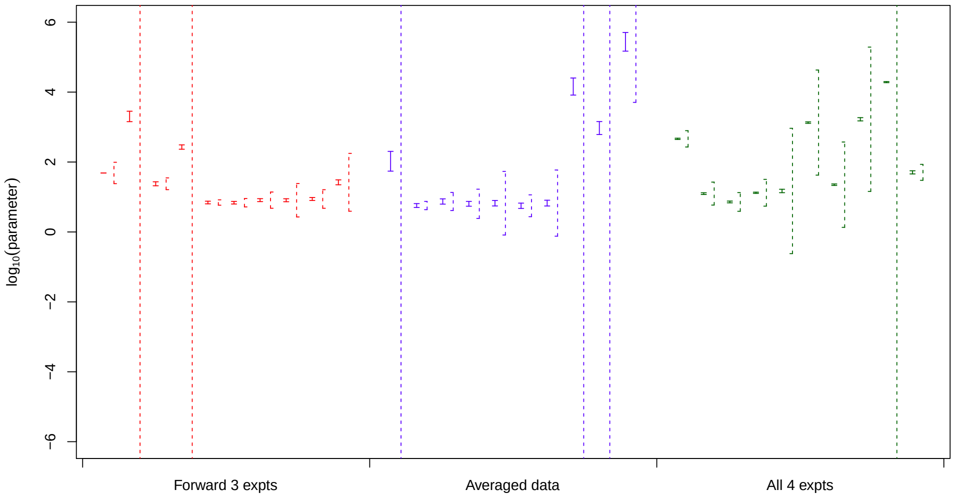

Figure S-5: Plot of dependent (solid lines) and independent (dashed lines) confidence intervals for the parameter V_{10}, for the ten best-scoring parameter sets from: a) (red) all three forward experiments, b) (blue) averaged data set, c) (green) all four experiments (3 forward, 1 reverse)

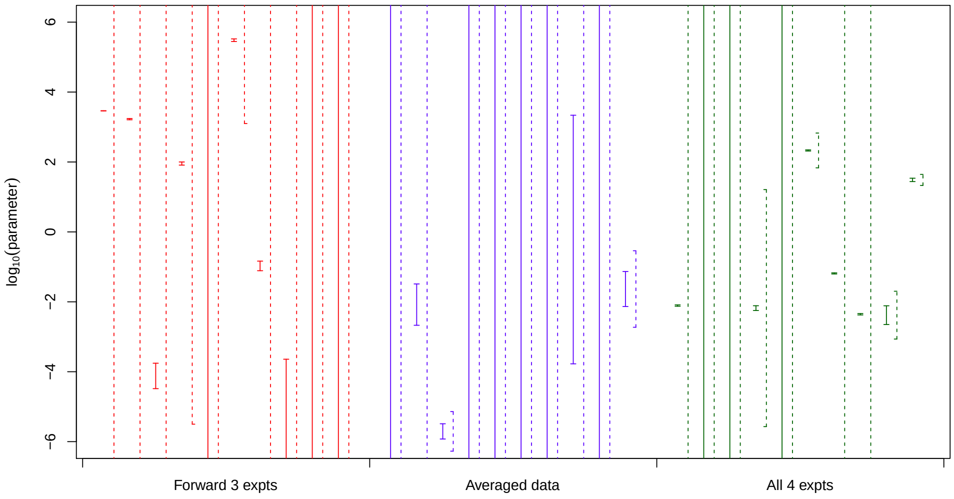

Figure S-6: Plot of dependent (solid lines) and independent (dashed lines) confidence intervals for the parameter K_1, for the ten best-scoring parameter sets from: a) (red) all three forward experiments, b) (blue) averaged data set, c) (green) all four experiments (3 forward, 1 reverse)

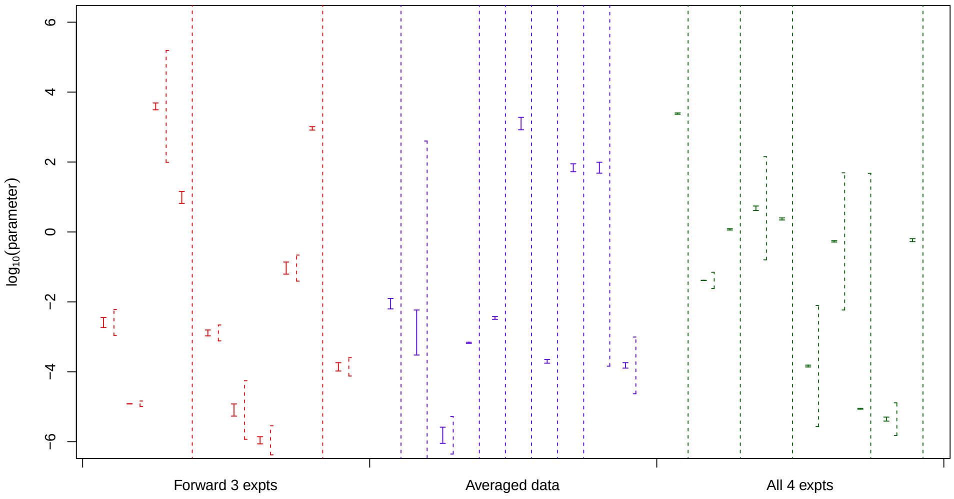

Figure S-7: Plot of dependent (solid lines) and independent (dashed lines) confidence intervals for the parameter K_2, for the ten best-scoring parameter sets from: a) (red) all three forward experiments, b) (blue) averaged data set, c) (green) all four experiments (3 forward, 1 reverse)

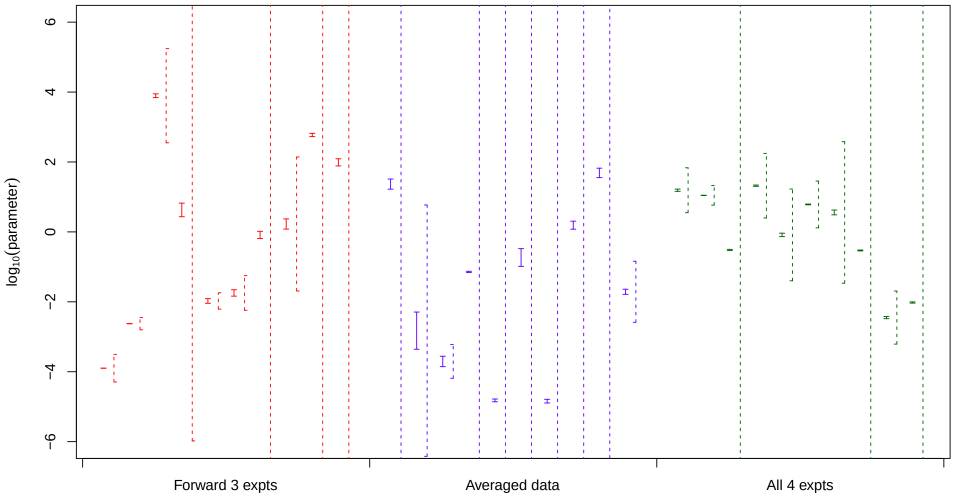

Figure S-8: Plot of dependent (solid lines) and independent (dashed lines) confidence intervals for the parameter K_4, for the ten best-scoring parameter sets from: a) (red) all three forward experiments, b) (blue) averaged data set, c) (green) all four experiments (3 forward, 1 reverse)

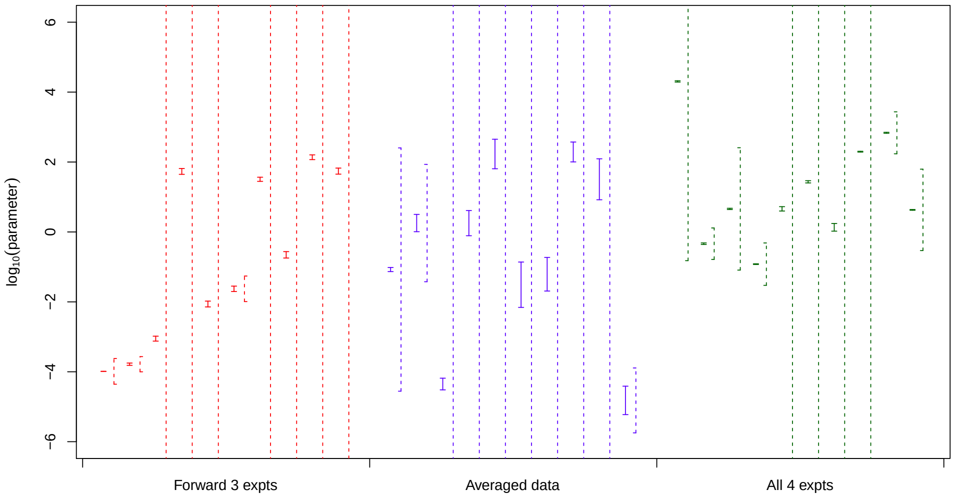

Figure S-9: Plot of dependent (solid lines) and independent (dashed lines) confidence intervals for the parameter K_8, for the ten best-scoring parameter sets from: a) (red) all three forward experiments, b) (blue) averaged data set, c) (green) all four experiments (3 forward, 1 reverse)

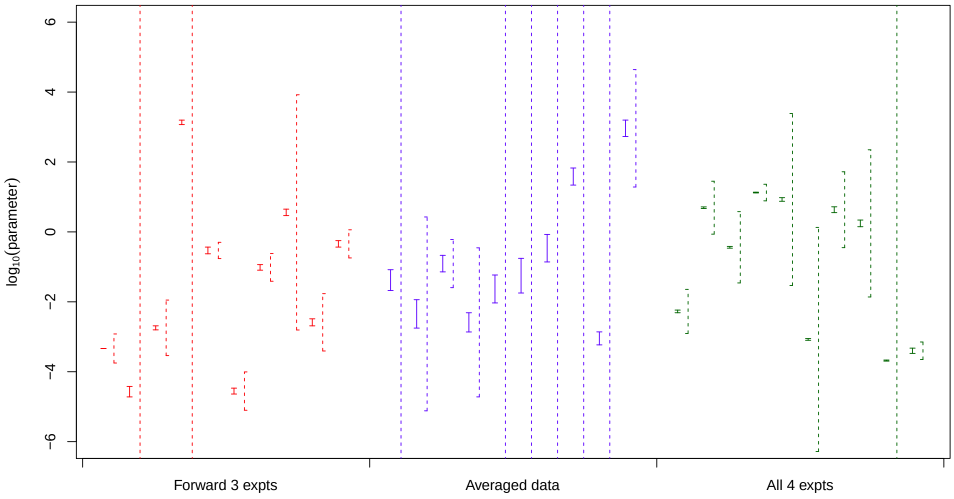

Figure S-10: Plot of dependent (solid lines) and independent (dashed lines) confidence intervals for the parameter K_{10}, for the ten best-scoring parameter sets from: a) (red) all three forward experiments, b) (blue) averaged data set, c) (green) all four experiments (3 forward, 1 reverse)

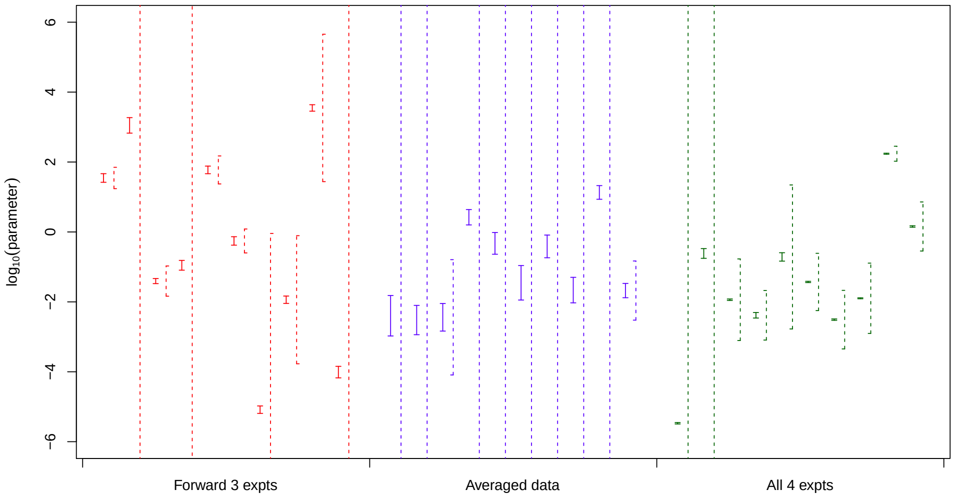

Figure S-11: Plot of dependent (solid lines) and independent (dashed lines) confidence intervals for the parameter α_3, for the ten best-scoring parameter sets from: a) (red) all three forward experiments, b) (blue) averaged data set, c) (green) all four experiments (3 forward, 1 reverse)

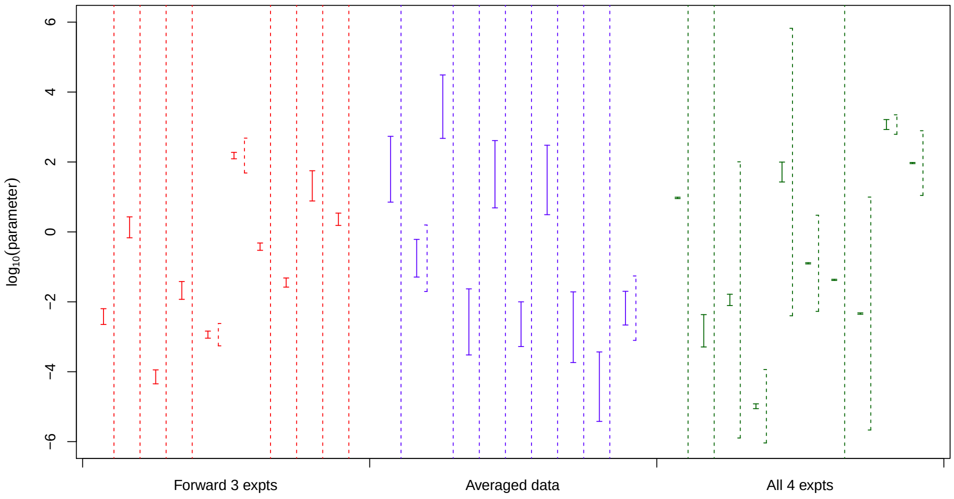

Figure S-12: Plot of dependent (solid lines) and independent (dashed lines) confidence intervals for the parameter α_5, for the ten best-scoring parameter sets from: a) (red) all three forward experiments, b) (blue) averaged data set, c) (green) all four experiments (3 forward, 1 reverse)

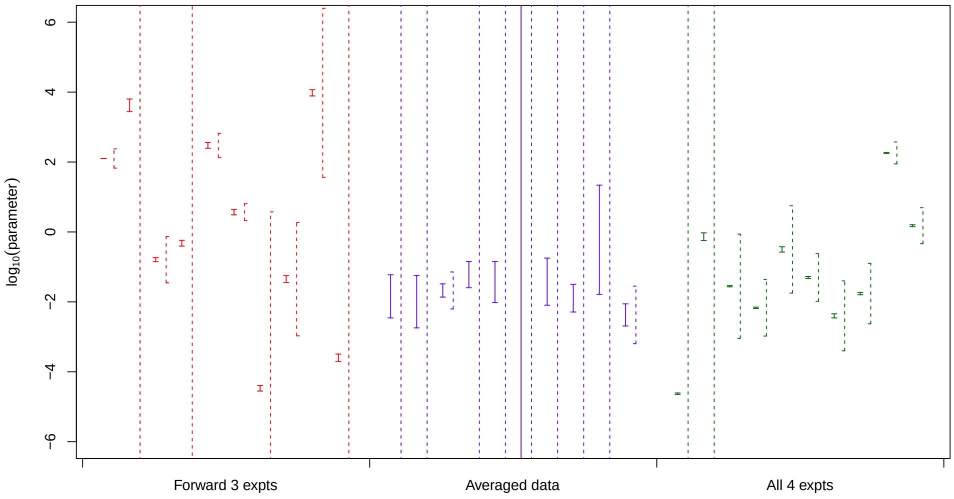

Figure S-13: Plot of dependent (solid lines) and independent (dashed lines) confidence intervals for the parameter α_6, for the ten best-scoring parameter sets from: a) (red) all three forward experiments, b) (blue) averaged data set, c) (green) all four experiments (3 forward, 1 reverse)

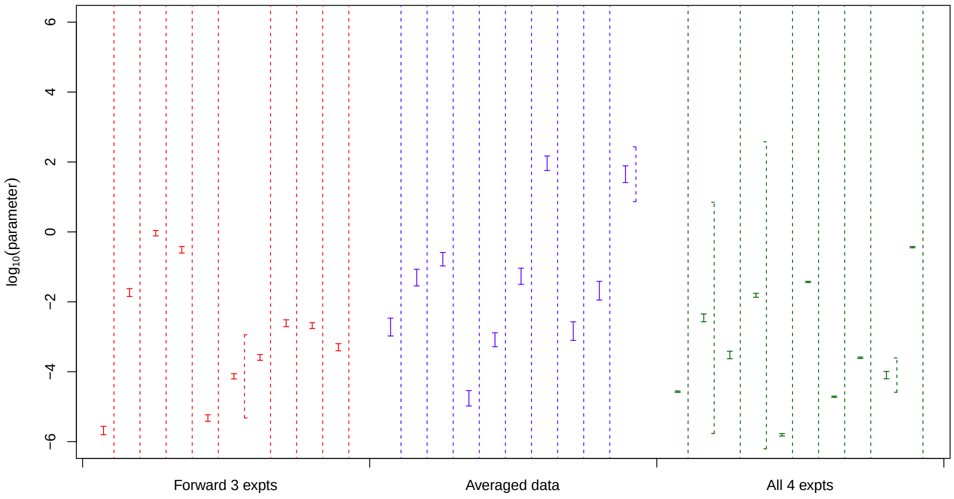

Figure S-14: Plot of dependent (solid lines) and independent (dashed lines) confidence intervals for the parameter α_7, for the ten best-scoring parameter sets from: a) (red) all three forward experiments, b) (blue) averaged data set, c) (green) all four experiments (3 forward, 1 reverse)

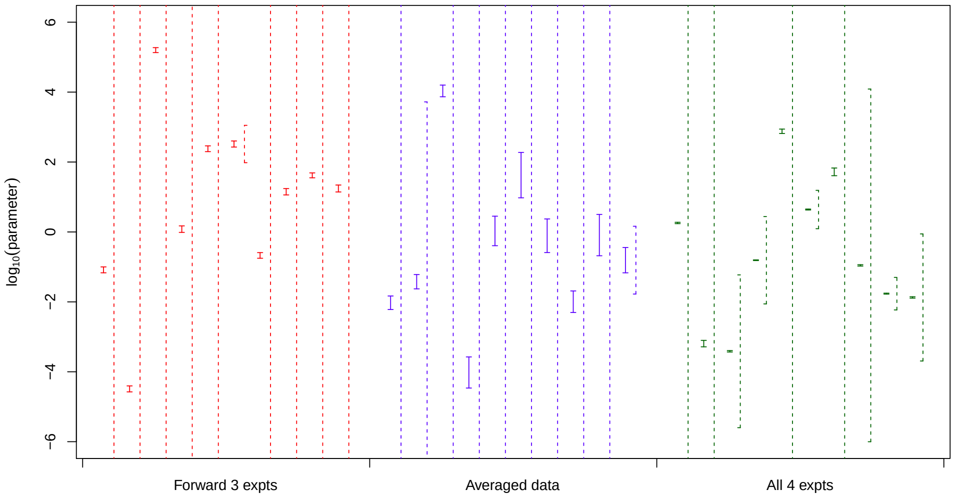

Figure S-15: Plot of dependent (solid lines) and independent (dashed lines) confidence intervals for the parameter α_9, for the ten best-scoring parameter sets from: a) (red) all three forward experiments, b) (blue) averaged data set, c) (green) all four experiments (3 forward, 1 reverse)

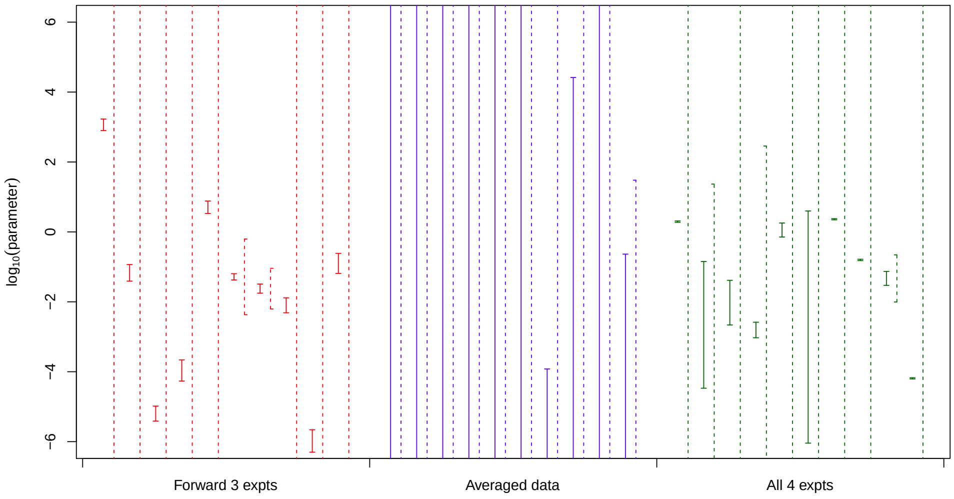

Figure S-16: Plot of dependent (solid lines) and independent (dashed lines) confidence intervals for the parameter r_{Ad}, for the ten best-scoring parameter sets from: a) (red) all three forward experiments, b) (blue) averaged data set, c) (green) all four experiments (3 forward, 1 reverse)

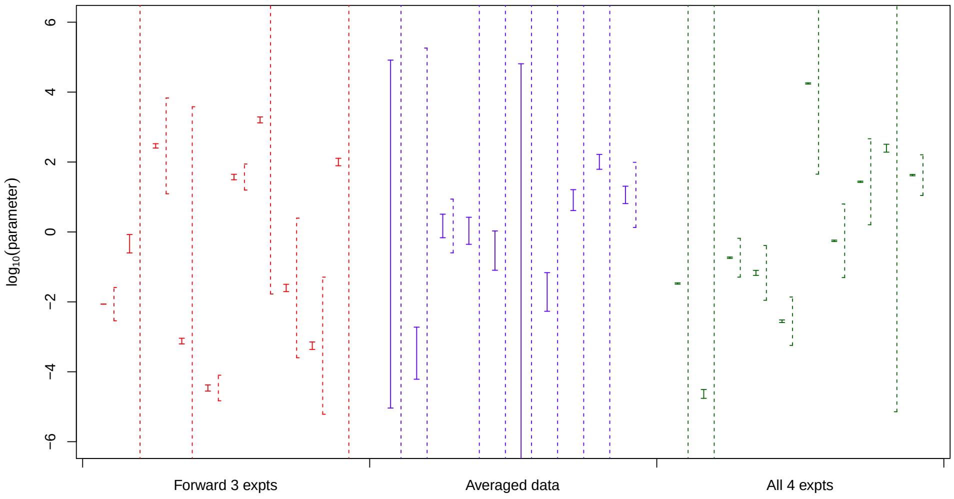

Figure S-17: Plot of dependent (solid lines) and independent (dashed lines) confidence intervals for the parameter r_{Cf}, for the ten best-scoring parameter sets from: a) (red) all three forward experiments, b) (blue) averaged data set, c) (green) all four experiments (3 forward, 1 reverse)

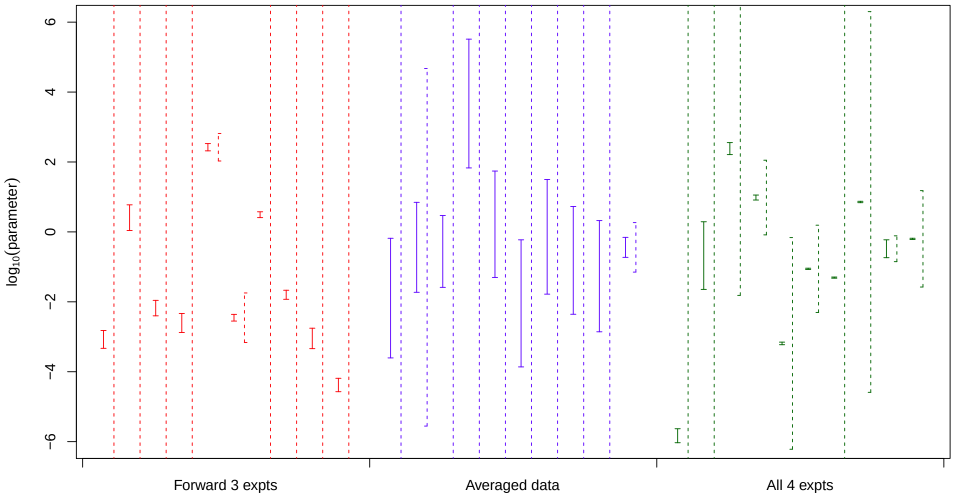

Figure S-18: Plot of dependent (solid lines) and independent (dashed lines) confidence intervals for the parameter r_{Ah}, for the ten best-scoring parameter sets from: a) (red) all three forward experiments, b) (blue) averaged data set, c) (green) all four experiments (3 forward, 1 reverse)

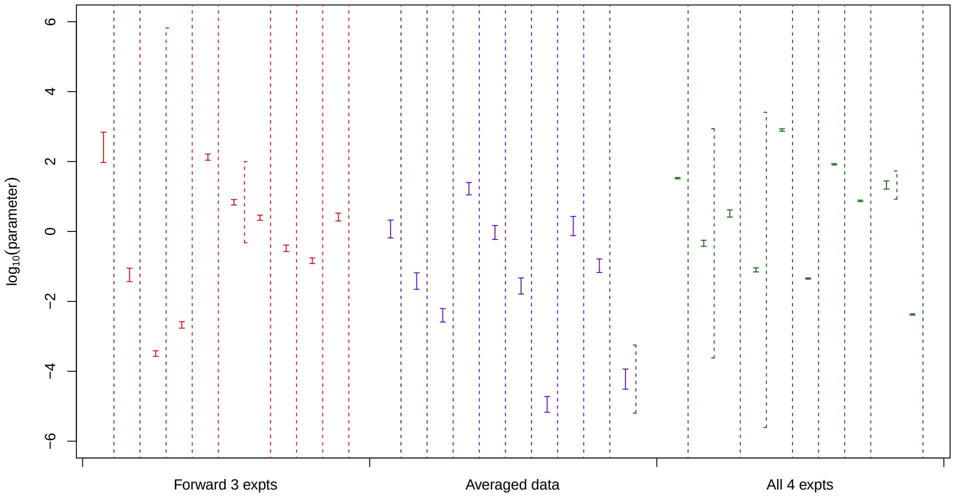

Figure S-19: Plot of dependent (solid lines) and independent (dashed lines) confidence intervals for the parameter r^+_{Ad}, for the ten best-scoring parameter sets from: a) (red) all three forward experiments, b) (blue) averaged data set, c) (green) all four experiments (3 forward, 1 reverse)

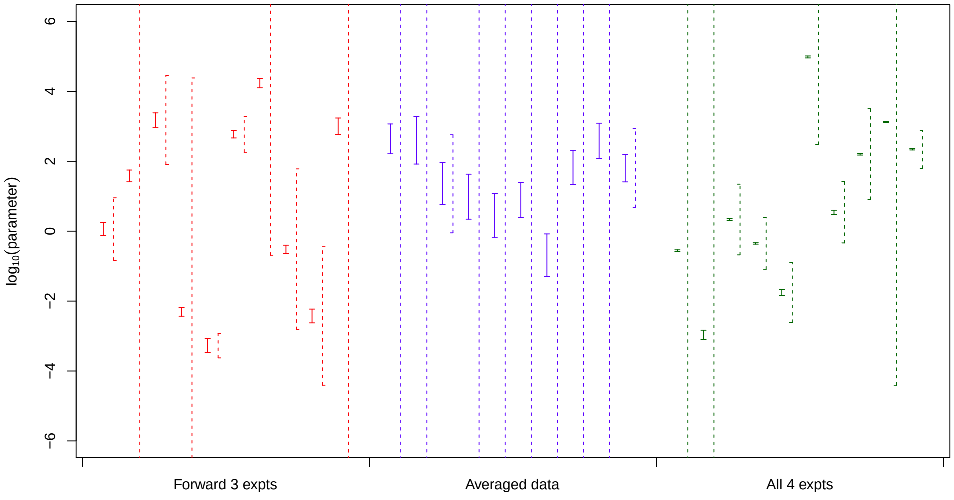

Figure S-20: Plot of dependent (solid lines) and independent (dashed lines) confidence intervals for the parameter r^+_{Cf}, for the ten best-scoring parameter sets from: a) (red) all three forward experiments, b) (blue) averaged data set, c) (green) all four experiments (3 forward, 1 reverse)

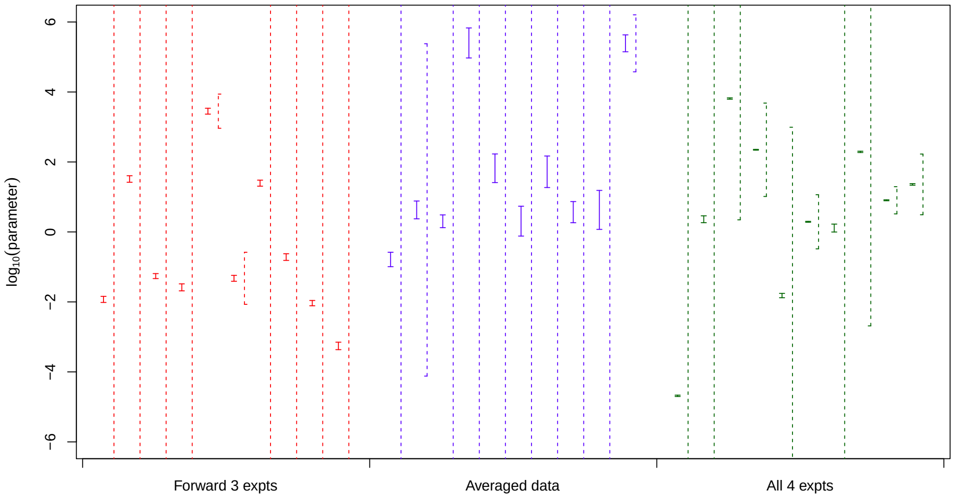

Figure S-21: Plot of dependent (solid lines) and independent (dashed lines) confidence intervals for the parameter r^+_{Ah}, for the ten best-scoring parameter sets from: a) (red) all three forward experiments, b) (blue) averaged data set, c) (green) all four experiments (3 forward, 1 reverse)

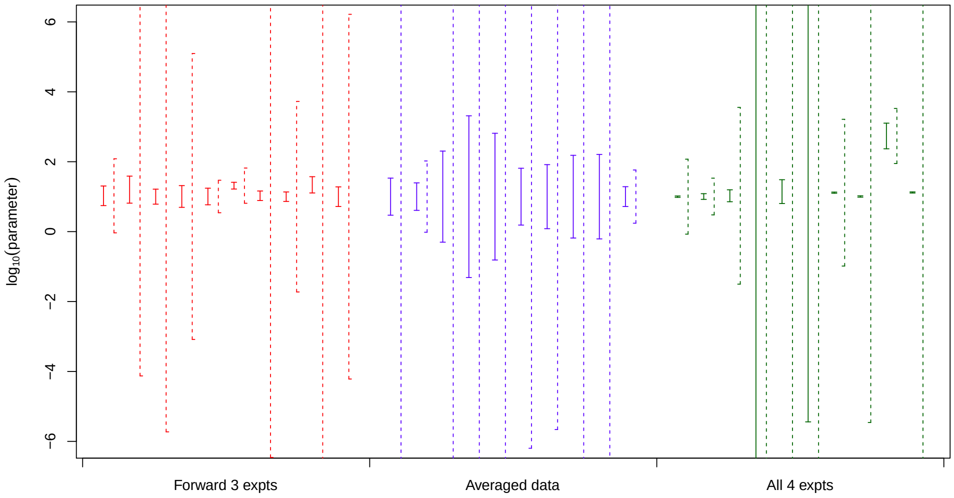

Figure S-22: Plot of dependent (solid lines) and independent (dashed lines) confidence intervals for the parameter n, for the ten best-scoring parameter sets from: a) (red) all three forward experiments, b) (blue) averaged data set, c) (green) all four experiments (3 forward, 1 reverse)

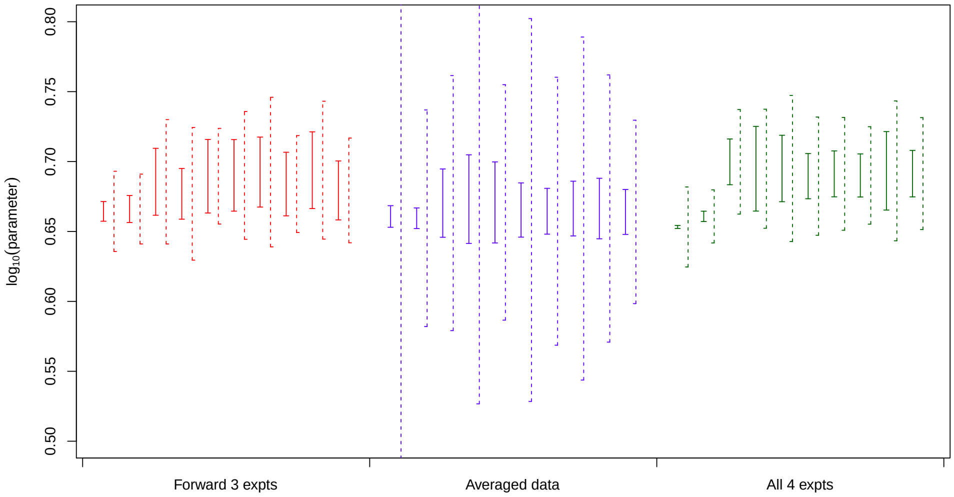

Figure S-23: Plot of dependent (solid lines) and independent (dashed lines) confidence intervals for the parameter p^*, for the ten best-scoring parameter sets from: a) (red) all three forward experiments, b) (blue) averaged data set, c) (green) all four experiments (3 forward, 1 reverse)

The average correlation plots of the correlation between parameters for the three ways of organising the experimental data are shown in figures S-24–S-26: the average of the three is shown in figure S-27,

References

[1] M. Ashyraliyev, J. Jaeger, and J. Blom. Parameter estimation and determinability analysis applied to Drosophila gap gene circuits. *BMC Systems Biology*, 2, 2008.

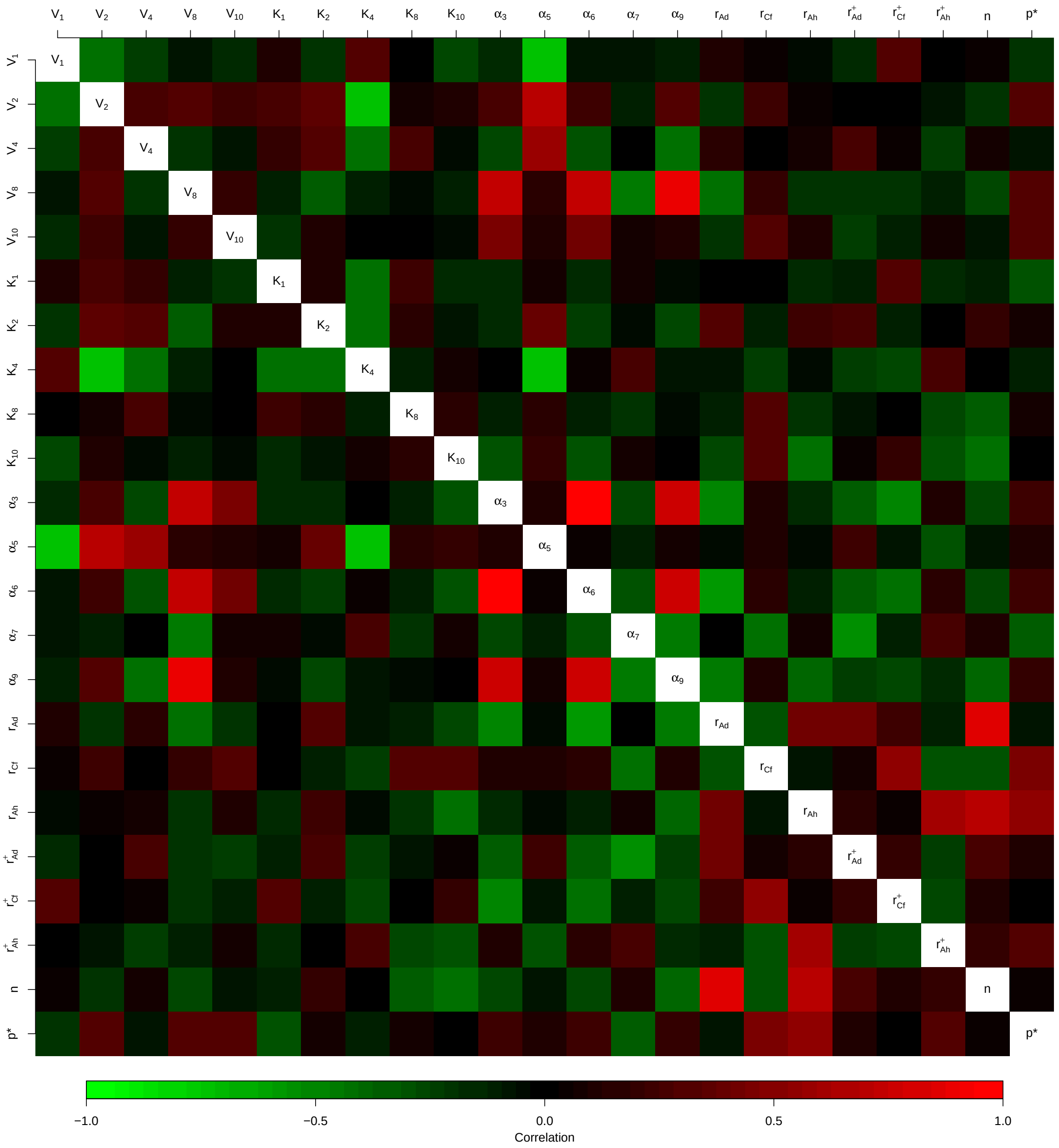

Figure S-24: Averaged correlation matrix between the parameters of the lowest 25 scoring parameter sets estimated using the three forward experiments.

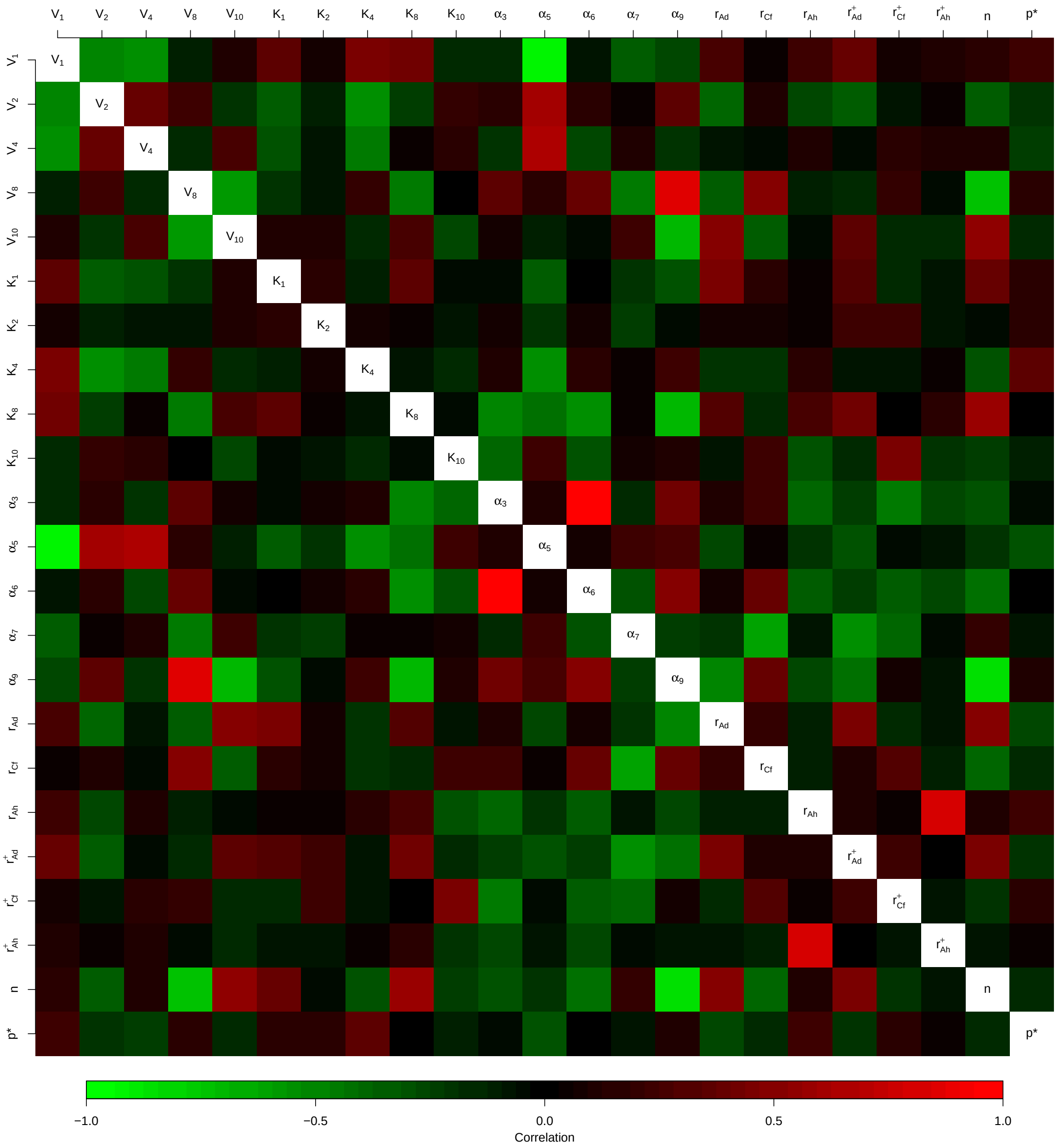

Figure S-25: Averaged correlation matrix between the parameters of the lowest 25 scoring parameter sets estimated using the averaged dataset.

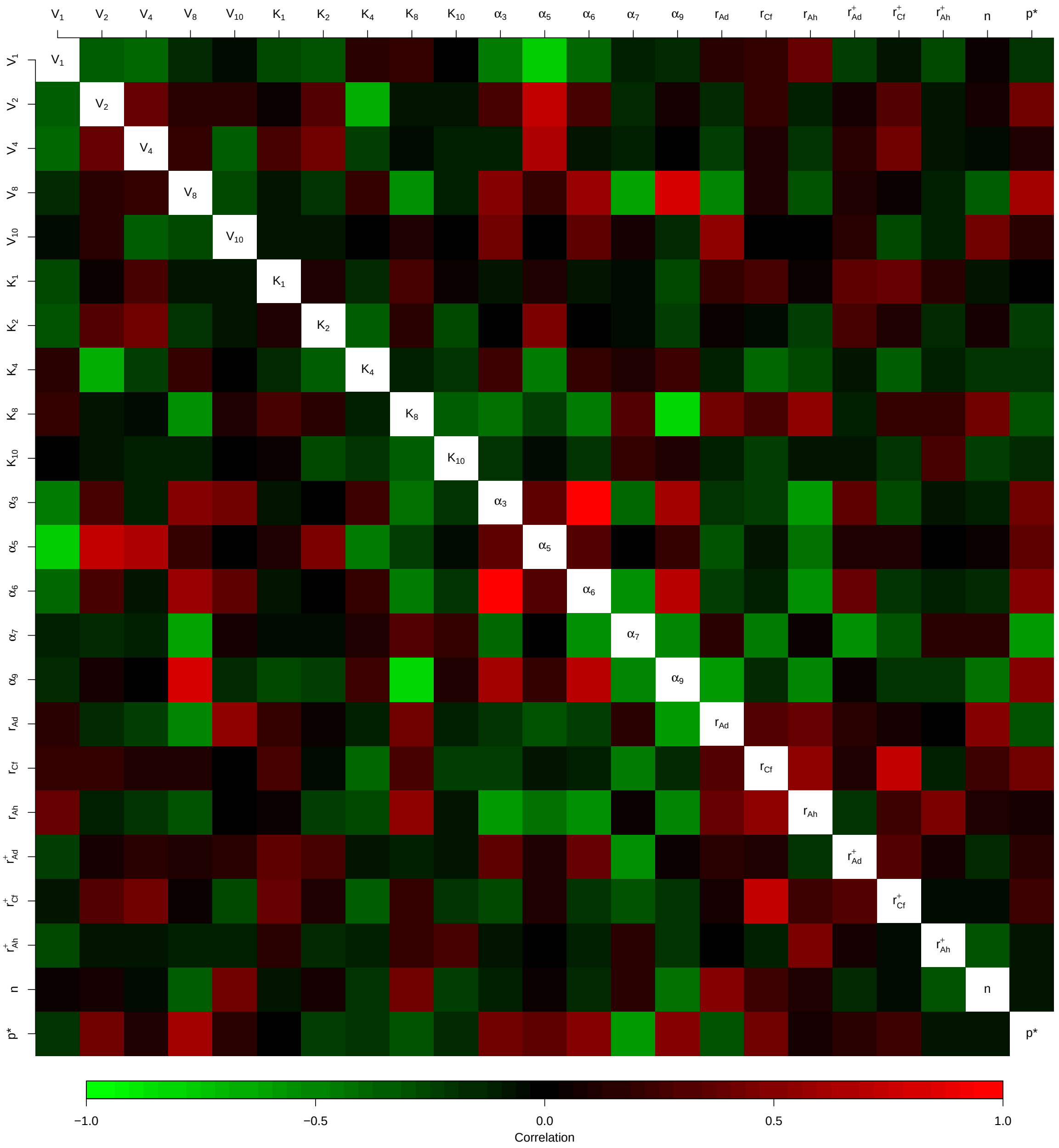

Figure S-26: Averaged correlation matrix between the parameters of the lowest 25 scoring parameter sets estimated using all four experimental datasets.

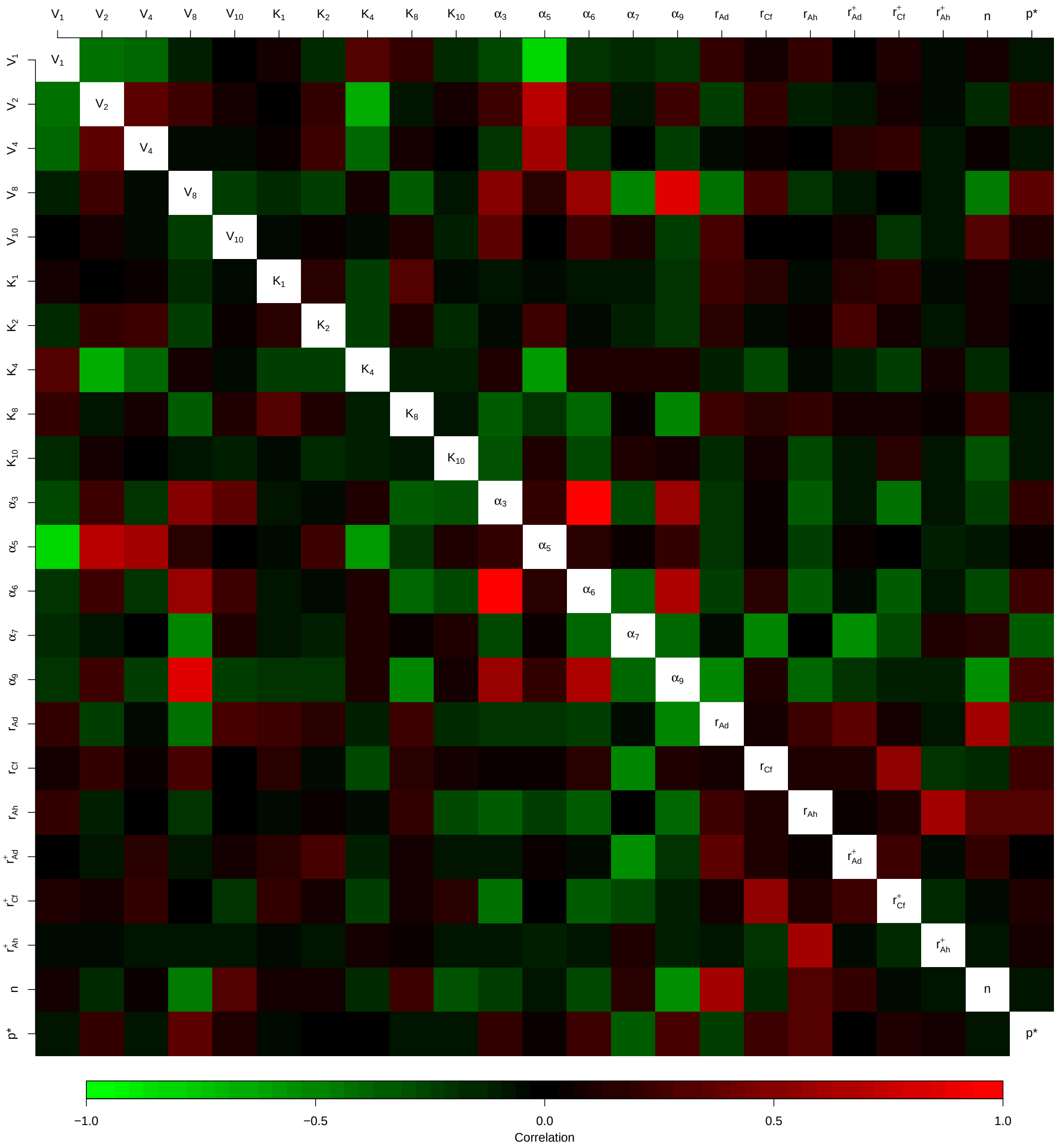

Figure S-27: Plot of average of figures S-24–S-26, the averaged correlation matrix of average of 25 lowest-scoring parameter sets for each dataset.
